# Bradyrhizobium diazoefficiens Requires Chemical Chaperones To Cope with Osmotic Stress during Soybean Infection

**DOI:** 10.1128/mBio.00390-21

**Published:** 2021-03-30

**Authors:** Raphael Ledermann, Barbara Emmenegger, Jean-Malo Couzigou, Nicola Zamboni, Patrick Kiefer, Julia A. Vorholt, Hans-Martin Fischer

**Affiliations:** a ETH Zurich, Institute of Microbiology, Zurich, Switzerland; b ETH Zurich, Institute of Molecular Systems Biology, Zurich, Switzerland; Mass General Hospital

**Keywords:** *Bradyrhizobium diazoefficiens*, compatible solutes, general stress response, nitrogen fixation, symbiosis, trehalose

## Abstract

When engaging in symbiosis with legume hosts, rhizobia are confronted with environmental changes, including nutrient availability and stress exposure. Genetic circuits allow responding to these environmental stimuli to optimize physiological adaptations during the switch from the free-living to the symbiotic life style. A pivotal regulatory system of the nitrogen-fixing soybean endosymbiont Bradyrhizobium diazoefficiens for efficient symbiosis is the general stress response (GSR), which relies on the alternative sigma factor σ^EcfG^. However, the GSR-controlled process required for symbiosis has not been identified. Here, we demonstrate that biosynthesis of trehalose is under GSR control, and mutants lacking the respective biosynthetic genes *otsA* and/or *otsB* phenocopy GSR-deficient mutants under symbiotic and selected free-living stress conditions. The role of trehalose as a cytoplasmic chemical chaperone and stress protectant can be functionally replaced in an *otsA* or *otsB* mutant by introducing heterologous genetic pathways for biosynthesis of the chemically unrelated compatible solutes glycine betaine and (hydroxy)ectoine. Alternatively, uptake of exogenously provided trehalose also restores efficient symbiosis and tolerance to hyperosmotic and hyperionic stress of *otsA* mutants. Hence, elevated cytoplasmic trehalose levels resulting from GSR-controlled biosynthesis are crucial for B. diazoefficiens cells to overcome adverse conditions during early stages of host infection and ensure synchronization with root nodule development.

## Abstract (precis)

The *Bradyrhizobium*-soybean symbiosis is of great agricultural significance and serves as a model system for fundamental research in bacterium-plant interactions. While detailed molecular insight is available about mutual recognition and early nodule organogenesis, our understanding of the host-imposed conditions and the physiology of infecting rhizobia during the transition from a free-living state in the rhizosphere to endosymbiotic bacteroids is currently limited.

## INTRODUCTION

Rhizobia are soil-inhabiting bacteria with the unique ability to form a mutualistic intracellular symbiosis with legume plants. When rhizobia encounter roots of host plants, they sense plant-derived flavonoids and respond by the synthesis of species-specific Nod factors which cause the induction of root-nodule primordia and root hair curling. Root hair-attached bacteria entrapped by curled root hairs multiply and form microcolonies from which they enter the root tissues via infection threads (ITs) guiding them to the cortical nodule primordium. Finally, bacteria are released from ITs into plant cells, where they differentiate into nitrogen-fixing bacteroids which provide fixed atmospheric dinitrogen in a bioavailable form to the host plant. In return, bacteroids receive reduced carbon sources and other nutrients from the host (see references [Bibr B1][Bibr B2][Bibr B5] and references therein).

Rhizobia are exposed to various types of stress conditions as free-living soil bacteria and during the transition to endosymbiotic bacteroids. In the soil, they may encounter temperature, nutrient starvation, desiccation, or pH stress while they must cope with host-imposed, transient defense responses during infection of root tissue (6, 7). Accordingly, rhizobia like many bacteria have evolved functions and mechanisms to withstand different stress types and alleviate the consequences of stress-induced cellular damages. These systems comprise chaperones, i.e., proteins that stabilize and protect proteins and other macromolecules (8–13), DNA repair systems (14), and reactive oxygen species (ROS)-detoxifying proteins (15–20) plus regulatory circuits to control the processes (21–25).

Apart from protein-based stress tolerance mechanisms, many bacteria rely on small molecules to protect macromolecules from damage. Collectively, these small molecules are termed compatible solutes (because they do not interfere with normal cellular functions) or chemical chaperones, which are considered panacea for protection of proteins and membranes from negative effects of a wide variety of stresses (26–29). Compatible solutes do not directly interact with macromolecules but stabilize their native conformation via preferential exclusion from and hydration of the macromolecule (see references 30 and 31 and references therein). Prominent compatible solutes comprise the quaternary amines ectoine, hydroxyectoine, and glycine betaine and the nonreducing disaccharide trehalose, composed of two α,α(1-1)-linked glucose residues. These molecules can accumulate to millimolar concentrations in stressed microorganisms either via uptake from the environment or by *de novo* synthesis (32, 33). Trehalose is a widespread multipurpose molecule which not only can counteract damaging effects of various stresses but also serves as carbon storage compound or signaling molecule (including some of its biosynthetic intermediates) (34–36). Unlike non-sugar-compatible solutes, trehalose also has membrane-protecting properties in addition to its protein-stabilizing function (37–39). Stress-induced *de novo* synthesis of trehalose is widespread in rhizobia; however, its exact role and involvement in symbiosis remained elusive so far (40–48).

In the soybean symbiont Bradyrhizobium diazoefficiens, mutants defective in the general stress response (GSR) display an aberrant symbiotic phenotype (49). The phenotype of the mutants manifests itself early on in symbiosis as reduced and delayed infection thread formation, which impedes proper nodule formation. Furthermore, GSR mutants lack competitiveness even when they are coinoculated with the wild type at a 10,000-fold excess. In agreement with these findings, we have shown that the GSR of B. diazoefficiens is specifically activated in microcolonies and ITs (50). The GSR in alphaproteobacteria is regulated by a partner switch mechanism involving the extracytoplasmic function (ECF) σ factor σ^EcfG^, its cognate anti-σ factor NepR and the anti-σ factor antagonist PhyR (for reviews, see references 51 and 52). Upon stress-induced activation, σ^EcfG^ redirects transcription to more than 100 genes (49). However, so far it remained unknown, which of the σ^EcfG^-controlled genes contribute to symbiotic proficiency or provide tolerance against free-living stresses. In the present study, we demonstrate that misregulated trehalose biosynthesis explains the symbiotic phenotype of GSR mutants. We further show that trehalose functions as a chemical chaperone to overcome stress-inducing conditions during the highly competitive early infection stage.

## RESULTS

### *B. diazoefficiens* mutants lacking the trehalose-6-phosphate pathway phenocopy the symbiotic defect of a Δ*ecfG* mutant.

**FIG 1:**
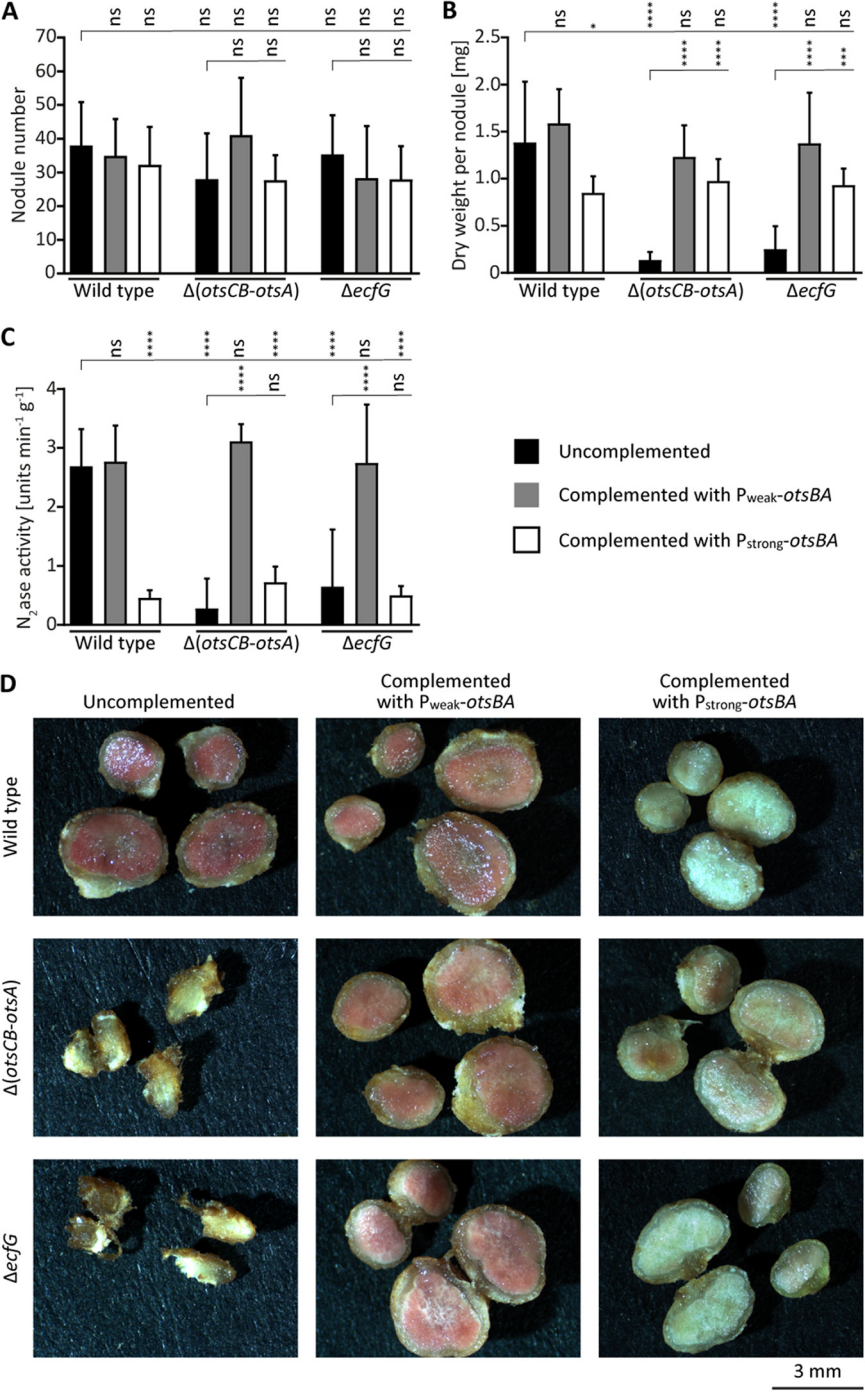
Constitutive trehalose biosynthesis via the trehalose-6-phosphate pathway complements the early symbiotic defects of a Δ*ecfG* mutant but expression levels are crucial for nitrogen fixation. Cells of wild-type *B. diazoefficiens* (strain 110*spc*4) and Δ(*otsCB-otsA*) (strain 9871) and Δ*ecfG* mutant strains (strain 8404) lacking or harboring *otsBA* ectopically expressed from a weak (P_weak_-*otsBA*; strains 1687, 71-1687, and 8404-1687) or strong promoter (P_strong_-*otsBA*; strains otsBA-1, 71-otsBA-1, and 8404-otsBA-1) were inoculated on soybean seedlings. (A to C) Plants were evaluated 21 days postinoculation (dpi) for nodule number (A), dry weight per nodule (B), and nitrogenase activity measured by acetylene reduction (C). (D) Cross sections of representative nodules showing the overall nodule morphology. Reddish or greenish-whitish color indicates the presence or absence of leghemoglobin, respectively. For panels A to C, the displayed values are means plus standard deviations (SD) (error bars) (*n* = 10). Statistical significances of pairwise comparisons made between columns marked with a vertical tick and adjacent columns under horizontal lines were determined using one-way analysis of variance (ANOVA) with Šidák multiple comparison correction and indicated as follows: ns, not significant (*P* ≥ 0.05); *, *P ≤ *0.05; ***, *P ≤ *0.001; ****, *P ≤ *0.0001.

The σ^EcfG^ regulon comprises different genes related to trehalose metabolism (49). One of the σ^EcfG^-controlled loci encodes the trehalose-6-phosphate (T6P) pathway of trehalose biosynthesis, which uses T6P synthase (TPS or OtsA) to condense UDP-glucose (UDP-Glc) and glucose-6-phosphate (Glc6P) to T6P. T6P phosphatase (TPP or OtsB) catalyzes the conversion of T6P to trehalose and inorganic phosphate. To investigate the supposed role of the T6P pathway in symbiosis, we constructed three different deletion mutants in the respective genes, *otsA* and *otsB* (strains 9904, 9906_Sm, and 9871; see Fig. S1 in the supplemental material). Notably, mutants deleted for *otsB* (strain 9906_Sm) or *otsB* as well as *otsA* (strain 9871) both also lacked *otsC*, which is located upstream of *otsB* but not required for symbiosis (see below). We found that mutants lacking either OtsA (T6P synthase) and/or OtsB (T6P phosphatase) phenocopy the Δ*ecfG* mutant in symbiosis with soybean. Specifically, trehalose biosynthesis and Δ*ecfG* mutants shared delayed and reduced formation of infection threads, induction of aborted pseudonodules with reduced dry weight, and impaired nitrogen fixation (Fig. 1 and Fig. S2A to K) (49, 50).

10.1128/mBio.00390-21.2 FIG S1 Genetic map of three *B. diazoefficiens* loci encoding trehalose biosynthesis genes and genotypes of respective deletion mutants. Download FIG S1, PDF file, 0.2 MB. Copyright © 2021 Ledermann et al. 2021 Ledermann et al. https://creativecommons.org/licenses/by/4.0/ This content is distributed under the terms of the Creative Commons Attribution 4.0 International license.

10.1128/mBio.00390-21.3 FIG S2 Symbiotic phenotype of additional mutants in trehalose biosynthesis and related genes. Download FIG S2, PDF file, 0.2 MB. Copyright © 2021 Ledermann et al. 2021 Ledermann et al. https://creativecommons.org/licenses/by/4.0/ This content is distributed under the terms of the Creative Commons Attribution 4.0 International license.

To provide further evidence for a role of the T6P pathway in symbiosis, we cloned *otsB* and *otsA* as an artificial *otsBA* operon under the control of constitutive promoters and integrated the genetic complementation constructs into the chromosomes of the wild type and Δ*ecfG* and Δ(*otsCB-otsA*) mutants. To drive *otsBA* expression, we chose either the strong *aphII* promoter (P_strong_ [53]) or a weaker promoter derived from the *B. diazoefficiens groESL*_2_ operon (P_weak_ [13]). Both constructs rescued the aborted-nodule phenotype (Fig. 1D) of the complemented mutants. Notably, nitrogenase activity at the wild-type level was observed only upon complementation with the weakly expressed *otsBA* operon, while expression of *otsBA* via a strong promoter caused the formation of whitish-greenish nodules and resulted in a more than 10-fold reduction of nitrogen fixation, even when the construct was present in a wild-type background (Fig. 1).

While the weakly expressed *otsBA* genes restored symbiotic properties of the Δ*ecfG* and Δ(*otsCB-otsA*) mutants equally well when inoculated singly (Fig. 1A to C), the complemented strains differed in their competitiveness. When inoculated together on soybean seedlings, the complemented Δ(*otsCB-otsA*) mutant was always more abundant in the resulting nodules, regardless of the ratio between the strains in the inoculum. Thus, EcfG-controlled functions other than trehalose biosynthesis contribute to symbiotic performance under competitive conditions (Fig. S3).

10.1128/mBio.00390-21.4 FIG S3 Trehalose synthesis via the T6P pathway cannot fully restore symbiotic competitiveness and stress tolerance of the Δ*ecfG* mutant. Download FIG S3, PDF file, 2.1 MB. Copyright © 2021 Ledermann et al. 2021 Ledermann et al. https://creativecommons.org/licenses/by/4.0/ This content is distributed under the terms of the Creative Commons Attribution 4.0 International license.

Apart from the T6P pathway, two additional trehalose biosynthesis routes are encoded in the *B. diazoefficiens* genome. (i) The TS pathway is specified by trehalose synthase (TS or TreS) which catalyzes the reversible conversion of maltose to trehalose. The respective gene (blr6767 or *treS*) was also found among the σ^EcfG^-controlled genes (49) but not the putative *treS* paralog bll0902. (ii) In the TreY/TreZ pathway, maltodextrins are converted to maltooligosaccharyl-trehalose. Specifically, TreY catalyzes the conversion of the α(1-4) to an α,α(1-1) linkage at the reducing end of the substrate oligosaccharide. Subsequently, TreZ releases the distal trehalose residue from the rest of the oligosaccharide. In order to study the role of the two alternative trehalose biosynthesis pathways of *B. diazoefficiens*, we generated deletion mutants in the respective genes (Fig. S1B and C). Mutant strains lacking *treZY* (strain 9885), *treZY* and *treS* (strain 9864), or bll0902 (strain 9899) had a wild type-like symbiotic phenotype, indicating that only the T6P pathway for trehalose biosynthesis is crucial for symbiosis (Fig. S2L to R). Consistent with this conclusion, we found that GSR-inducing stress conditions triggered trehalose accumulation via the T6P pathway but not by either of the alternative pathways (Fig. S4).

10.1128/mBio.00390-21.5 FIG S4 Trehalose content of wild-type and mutant *B. diazoefficiens* cells grown under stressed and unstressed conditions. Download FIG S4, PDF file, 0.2 MB. Copyright © 2021 Ledermann et al. 2021 Ledermann et al. https://creativecommons.org/licenses/by/4.0/ This content is distributed under the terms of the Creative Commons Attribution 4.0 International license.

### Trehalose confers tolerance toward hyperosmotic and hyperionic stress.

The GSR in *B. diazoefficiens* regulates genes involved in tolerance against various unrelated stresses under free-living conditions (49). Since Δ*ecfG* and T6P pathway mutants both displayed the same phenotypes in symbiosis, we wondered whether they also share similar stress tolerance profiles.

**FIG 2:**
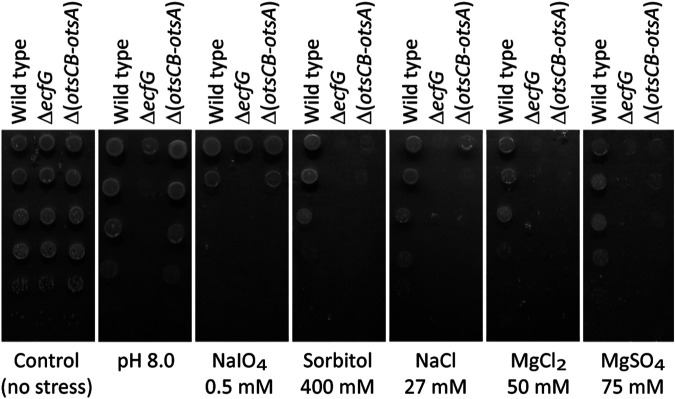
Reduced trehalose biosynthesis via the T6P pathway in a *B. diazoefficiens* Δ*ecfG* mutant accounts for increased sensitivity against ionic and nonionic osmostress, but not against alkaline or oxidative stress. Cell suspensions of the wild type (strain 110*spc*4), Δ*ecfG* mutant (strain 8404), and Δ(*otsCB-otsA*) mutant (strain 9871) were adjusted to an OD_600_ of 0.1, and 4 μl of 10-fold serial dilutions were spotted on V3C agar plates exposing cells to the indicated stress conditions.

We found that trehalose biosynthesis via the T6P pathway accounts for tolerance against hyperosmotic conditions (400 mM sorbitol) and against various ionic stresses (27 mM NaCl, 50 mM MgCl_2_, 75 mM MgSO_4_), as the Δ(*otsCB-otsA*) mutant showed reduced viability under these stress conditions similar to the Δ*ecfG* mutant. However, a functional GSR conferred tolerance to additional stresses such as alkaline pH conditions (pH 8.0) or oxidative stress (0.5 mM NaIO_4_) in a T6P pathway-independent manner (Fig. 2).

### Exogenous trehalose can bypass the requirement for endogenous trehalose.

**FIG 3:**
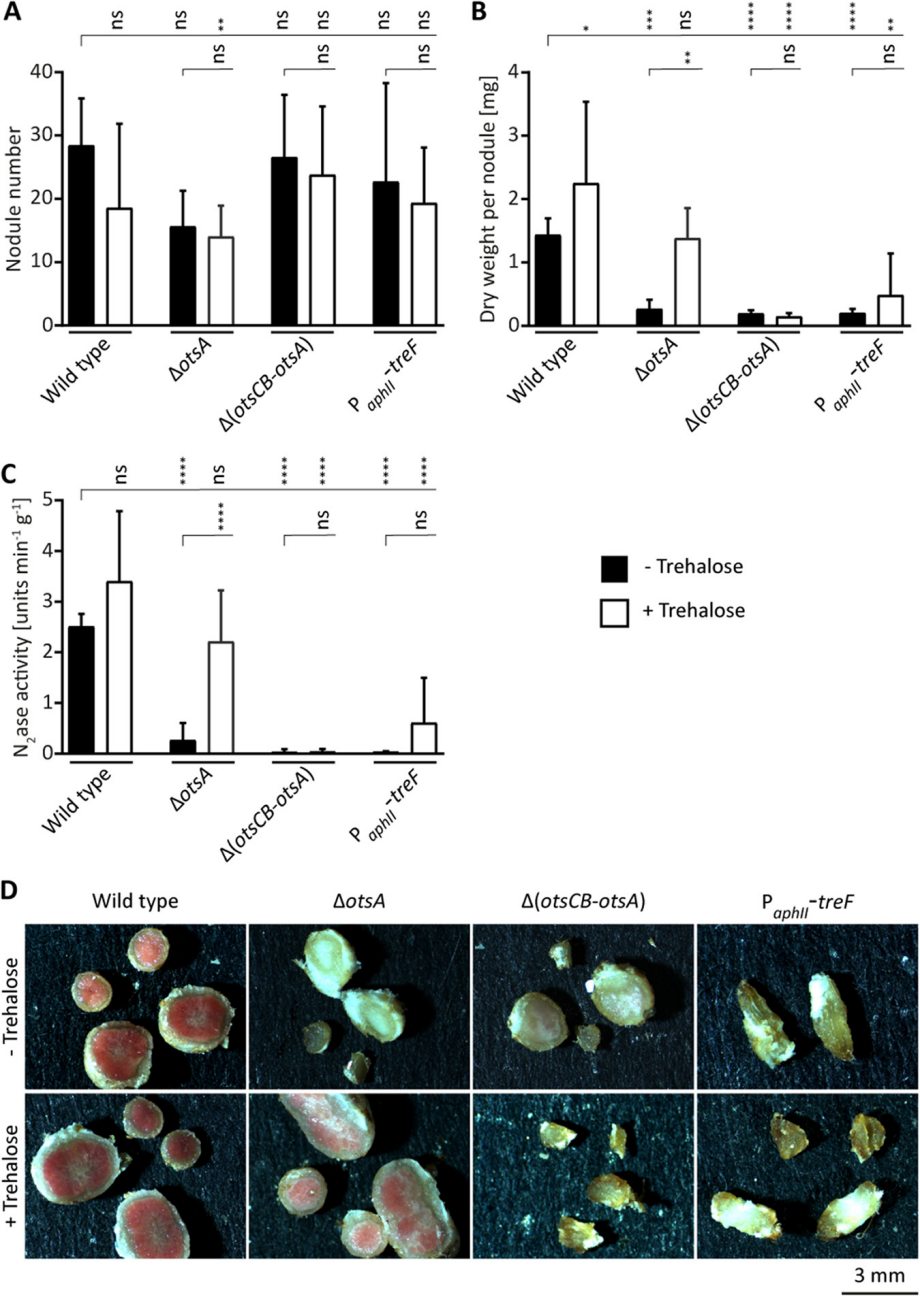
Exogenous trehalose can correct the symbiotic defect of a *B. diazoefficiens* Δ*otsA* mutant. Cells of the wild type (strain 110*spc*4), Δ*otsA* mutant (strain 9904), or Δ(*otsCB-otsA*) mutant (strain 9871), and strain TreF-1 expressing E. coli *treF* from the constitutive P*_aphII_* promoter in a wild-type background were inoculated on soybean seedlings. These strains were grown in 180-ml jars filled with vermiculite which was soaked with mineral salts solution and supplemented with 10 mmol of trehalose (for details, see Materials and Methods). (A to C) Plants were evaluated 21 dpi for nodule number (A), dry weight per nodule (B), and nitrogenase activity measured by acetylene reduction (C). (D) Cross sections of representative nodules show overall nodule morphology and presence of reddish color indicative for leghemoglobin. For panels A to C, the values displayed are means and error bars represent standard deviations (SD) (*n* ≥ 8). Statistical significances of pairwise comparisons made between columns marked with a vertical tick and adjacent columns under horizontal lines were determined using one-way ANOVA with Šidák multiple comparison correction and indicated as follows: ns, not significant (*P* ≥ 0.05); *, *P ≤ *0.05; **, *P ≤ *0.01; ***, *P ≤ *0.001; ****, *P ≤ *0.0001. Note that the data shown in this figure were generated in the same experiment underlying the larger data set shown in Fig. S5B to E in the supplemental material. Hence, the same data are shown for the reference conditions (wild type and Δ*otsA* mutant, with and without trehalose) in both figures. Statistical analysis was performed on the entire data set.

To test whether intracellular trehalose is required for symbiotic proficiency, we constructed *B. diazoefficiens* strain TreF-1 which constitutively expresses the Escherichia coli gene for a cytoplasmic trehalase (P*_aphII_-treF*) in a wild-type background. We determined trehalose content of stressed and unstressed wild-type and different mutant cells (Fig. S4). Congruent with the trehalose-hydrolyzing activity of TreF and unlike the wild type, *treZY*, *treS treZY*, or bll0902 mutants, we found that stressed cells of strain TreF-1 did not accumulate trehalose, and likewise mutants lacking the GSR (Δ*ecfG*) or the T6P pathway [Δ*otsA*, Δ*otsCB*, or Δ(*otsCB-otsA*)]. Moreover, strain TreF-1 phenocopied the latter group of mutants both in symbiosis and under free-living osmostress conditions (Fig. 3 and Fig. S5A).

10.1128/mBio.00390-21.6 FIG S5 Exogenous trehalose can rescue a Δ*otsA* mutant under hyperosmotic conditions and in symbiosis. Download FIG S5, PDF file, 0.8 MB. Copyright © 2021 Ledermann et al. 2021 Ledermann et al. https://creativecommons.org/licenses/by/4.0/ This content is distributed under the terms of the Creative Commons Attribution 4.0 International license.

Next, we examined how the addition of exogenous trehalose to the plant growth medium would affect mutants lacking the T6P pathway. When we inoculated plants with the Δ*otsA* mutant strain 9904 and added exogenous trehalose to the plant growth medium, the aborted-nodule phenotype was suppressed and normal nodules were formed (Fig. 3). Under these conditions, the mutant induced leghemoglobin-containing nodules that were similar to wild-type nodules with respect to dry weight and nitrogenase activity. However, strain TreF-1 expressing E. coli *treF* could not be rescued by exogenous trehalose (Fig. 3). Complementation was specific for trehalose, as neither the addition of sucrose (another nonreducing disaccharide) or glucose (the monomer of the trehalose homodisaccharide) rescued the Δ*otsA* mutant (Fig. S5B to E). In fact, addition of sucrose exacerbated the symbiotic defect of the Δ*otsA* mutant such that barely any nodules were formed. Since the wild type was not affected by the addition of sucrose, we speculate that the Δ*otsA* mutant was more sensitive to the increased osmotic stress imposed by the exogenous sucrose.

The *otsC* gene present in the *otsCB* operon encodes a putative major facilitator superfamily (MFS)-type sugar transporter (Fig. S6). A markerless *otsC* in-frame deletion leaving *otsB* intact (Fig. S1A) had no effect on symbiosis (Fig. S2F to J). Given that *otsC* is coexpressed with *otsB* and annotated as a sugar transporter, we speculated that *otsC* might be involved in trehalose uptake. Indeed, when we inoculated the Δ(*otsCB-otsA*) mutant strain 9871 on soybean plants and added exogenous trehalose, the mutant was not rescued (Fig. 3). We also attempted to complement different *ots* mutants by exogenous trehalose added to free-living cells growing on sorbitol-containing agar medium, which evokes hyperosmotic stress conditions. While the Δ*otsA* mutant was rescued to almost wild-type stress tolerance, trehalose did not enhance osmotolerance of the Δ(*otsCB-otsA*) and Δ*otsCB* mutants. In the same experiment, neither the *treF*-expressing strain TreF-1 nor the Δ*ecfG* mutant 8404, which is unable to induce σ^EcfG^-dependent *otsCB* expression (49), was rescued by exogenous trehalose (Fig. S5A). Taken together, these results support that OtsC is a trehalose transporter and further document the intracellular requirement of trehalose as stress protectant.

10.1128/mBio.00390-21.7 FIG S6 Transcriptional organization and regulation of the *B. diazoefficiens otsCB-otsA* gene region. Download FIG S6, PDF file, 0.2 MB. Copyright © 2021 Ledermann et al. 2021 Ledermann et al. https://creativecommons.org/licenses/by/4.0/ This content is distributed under the terms of the Creative Commons Attribution 4.0 International license.

### Trehalose can be functionally replaced by other chemical chaperones.

Given our findings that (i) trehalose accounts for osmostress tolerance but not other stress tolerances mediated by the GSR and (ii) trehalose is needed intracellularly in *B. diazoefficiens* during symbiosis, we concluded that trehalose mediates tolerance to an osmotic stress during the infection process. We postulated that this role of trehalose as a chemical chaperone can be functionally replaced by structurally unrelated osmoprotectants. To test our hypothesis, we cloned genes involved in the biosynthesis of glycine betaine and ectoine/hydroxyectoine, which were shown to confer osmoprotection when heterologously expressed in E. coli (54, 55), in a synthetic operon (*gsmT-sdmT-metK2-ectA-ectB-ectC-ectD-ask*). The construct (hereafter referred to as GSMABCDA) was chromosomally integrated into the wild type and the Δ(*otsCB-otsA*) mutant such that it was transcribed from the endogenous σ^EcfG^-dependent promoter of the bll6649 gene (50). Glycine betaine biosynthesis genes *gsmT*, *sdmT*, and *metK2* originated from the halophilic bacterium Halorhodospira halochloris and encode the bifunctional glycine/sarcosine *N*-methyltransferase GsmT, the bifunctional sarcosine/dimethylglycine *N*-methyltransferase SdmT, and the *S*-adenosylmethionine (SAM)-regenerating enzyme *S*-adenosylmethionine synthetase MetK2, respectively (55–57). Genes *ectABCD* and *ask* of Pseudomonas stutzeri specify ectoine/hydroxyectoine biosynthesis and code for l-2,4-diaminobutyric acid acetyltransferase (EctA), diaminobutyrate-2-oxoglutarate transaminase (EctB), l-ectoine synthase (EctC), ectoine hydroxylase (EctD), and a feedback-insensitive aspartate kinase (Ask), respectively (54, 58) (see Fig. S7A to C).

10.1128/mBio.00390-21.8 FIG S7 Complementation of trehalose biosynthesis mutants by expression of recombinant genes encoding biosynthesis for different chemical chaperones. Download FIG S7, PDF file, 1.0 MB. Copyright © 2021 Ledermann et al. 2021 Ledermann et al. https://creativecommons.org/licenses/by/4.0/ This content is distributed under the terms of the Creative Commons Attribution 4.0 International license.

**FIG 4:**
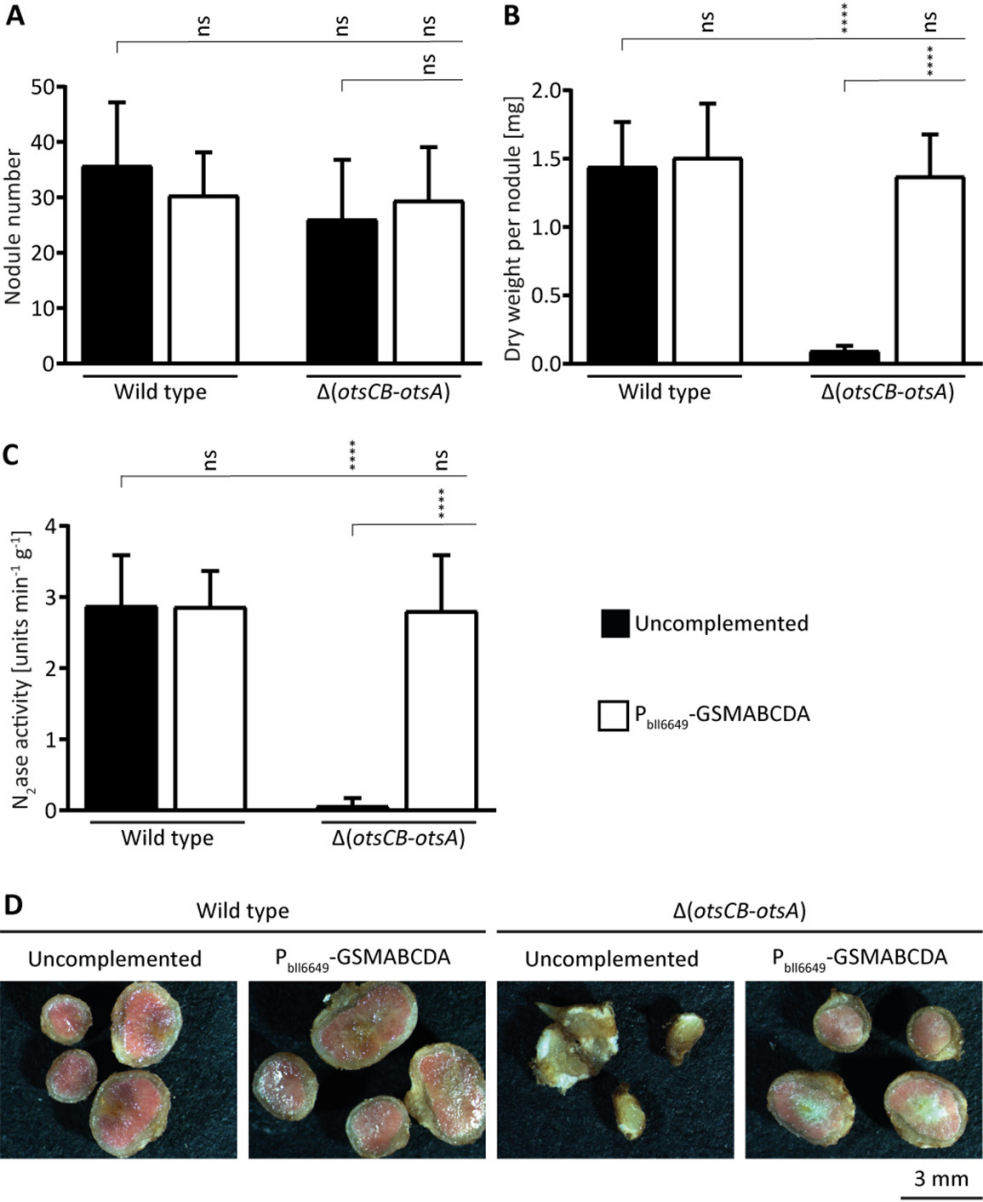
Endogenous synthesis of chemically unrelated osmoprotectants can replace the symbiotic need for intracellular trehalose. Cells of wild-type *B. diazoefficiens* (strain 110*spc*4), Δ(*otsCB-otsA*) mutant (strain 9871), and both strains expressing a heterologous synthetic operon (GSMABCDA) for the biosynthesis of glycine betaine (from Halorhodospira halochloris DSM 1059) and hydroxyectoine (from Pseudomonas stutzeri ATCC 17588) under the control of the endogenous σ^EcfG^-dependent bll6649 promoter (P_bll6649_; strains 9987 and 71-87, respectively) were inoculated on soybean seedlings. (A to C) Plants were evaluated 21 dpi for nodule number (A), dry weight per nodule (B), and nitrogenase activity measured by acetylene reduction (C). (D) Cross sections of representative nodules show overall nodule morphology and presence of red interior color indicative for leghemoglobin). For panels A to C, the values displayed are means and error bars represent SD (*n* = 10). Statistical significances of pairwise comparisons made between columns marked with a vertical tick and adjacent columns under horizontal lines were determined using one-way ANOVA with Šidák multiple comparison correction and indicated as follows: ns, not significant (*P* ≥ 0.05); ****, *P ≤ *0.0001.

In extracts of the resulting strains [wild type plus GSMABCDA (strain 9987) and Δ(*otsCB-otsA*) plus GSMABCDA (strain 71-87)], ectoine and to a lesser extent, hydroxyectoine were readily detected by mass spectrometry analysis, but glycine betaine was present only in minute amounts and also found in the uncomplemented parental strains. Both recombinant strains did not differ in their trehalose content compared to the respective parental strains (Fig. S7D). Synthesis of (hydroxy)ectoine in the complemented Δ(*otsCB-otsA*) mutant restored salt stress tolerance and to some degree also tolerance against nonionic osmostress, whereas the wild type producing (hydroxy)ectoine showed no increased stress tolerance (Fig. S7E). Most notably, synthesis of ectoine/hydroxyectoine in the complemented Δ(*otsCB-otsA*) mutant restored symbiotic proficiency (Fig. 4). The spherical nodules had a pink interior, and their dry weight and nitrogenase activity did not differ from wild type-induced nodules. Taken together, these results indicate that impaired osmostress tolerance is sufficient to explain the strong symbiotic phenotype associated with the loss of the global regulator σ^EcfG^.

## DISCUSSION

The extracytoplasmic function sigma factor σ^EcfG^ is crucial for efficient host infection and nitrogen fixation. Its regulon comprises more than a hundred genes. It was previously unknown whether a large part of the regulon or only a single locus is responsible for this conspicuous phenotype (49, 50). In the present study, we assigned the symbiotic relevance of σ^EcfG^ to a sole defined cellular function, trehalose biosynthesis via the T6P pathway. Mutants lacking either the T6P pathway or the GSR induced both similar, aborted pseudonodules in symbiosis with soybean and displayed reduced osmostress tolerance. However, other GSR-controlled cellular functions additionally contribute to nodulation competitiveness.

Among mono- and disaccharides, trehalose is the predominant sugar in *B. diazoefficiens* cells grown in different media (45). It is further accumulated by induction of respective biosynthetic genes in stressed free-living cells or bacteroids of *B. diazoefficiens* and other rhizobial species (48, 59–61). It was reported that the specific activity of trehalose synthase and maltooligosyltrehalose synthase, the key enzymes of the TreS and TreY/TreZ pathways for trehalose biosynthesis, respectively, are upregulated and the T6P synthase downregulated in mature nodules when compared with free-living cells (42). Here we demonstrated that the latter pathway is indispensable at early infection stages of symbiosis. In line with findings by Sugawara et al. (48), we found that the TreS and TreY/TreZ pathways do not substantially contribute to the trehalose pool in infecting bacteria possibly because the concentration of substrates of the respective enzymes (maltose [TreS], maltodextrins [TreY], maltooligosyltrehalose [TreZ]) are low in those cells (42, 48). At present, no specific role can be attributed to the alternative pathways for trehalose biosynthesis though they are present and functional in numerous rhizobial species (40, 46). It was proposed that *B. diazoefficiens* TreS functions in degradation rather than synthesis of trehalose because increased levels of trehalose and lower levels of maltose were present in a *treS* mutant relative to the wild type (48).

In our previous work, we showed that the GSR is transiently activated in *B. diazoefficiens* at early infection stages but not in mature nitrogen-fixing bacteroids (50). This finding combined with the identification of trehalose biosynthesis via the T6P pathway as the symbiosis-relevant, GSR-controlled process implies a transient need of trehalose during the initial steps of host infection when conditions might be particularly unfavorable for invading bacteria. The transient role of trehalose during formation of symbiosis was further evidenced by our observation that part of the nodules induced by the Δ(*otsCB-otsA*) mutant showed a pink interior color which is indicative for leghemoglobin and developed considerable nitrogenase activity after prolonged plant growth (28 days postinoculation [dpi]; see Fig. S8 in the supplemental material) similar to what we described previously for *ecfG* and *phyR* mutants (49). Thus, TPS/TPP-deficient mutant bacteria have the potential to colonize nodule cells, proliferate, and synthesize the nitrogen fixation apparatus when they succeed in overcoming the infection bottleneck in microcolonies and ITs.

10.1128/mBio.00390-21.9 FIG S8 Symbiotic phenotype of wild type, Δ(*otsCB-otsA*), and Δ*ecfG* mutants 28 dpi. Download FIG S8, PDF file, 0.3 MB. Copyright © 2021 Ledermann et al. 2021 Ledermann et al. https://creativecommons.org/licenses/by/4.0/ This content is distributed under the terms of the Creative Commons Attribution 4.0 International license.

Our complementation experiments indicated that controlled trehalose synthesis via the T6P pathway is crucial for effective symbiosis. Constitutive, strong expression of *otsBA* genes in the Δ(*otsCB-otsA*) mutant restored only nodulation, but bacteroids inside these nodules had very low nitrogenase activity. Moreover, the same overexpression construct present in wild-type cells decreased nitrogenase activity to levels of the uncomplemented Δ(*otsCB-otsA*) mutant. Excess trehalose *per se* is probably not the reason for the negative effect of the deregulated T6P pathway in those cells, as trehalose can make up to 3% of the dry weight of free-living *B. diazoefficiens* cells (45). We propose that unregulated overexpression of *otsBA* causes a metabolic imbalance, which may lead to energy deficiency and interference with the energy-demanding nitrogenase reaction.

Trehalose is a very versatile molecule for which a plethora of functions have been reported for diverse organisms from all kingdoms of life (62). While it can serve as a carbon source or storage compound in many bacteria, *B. diazoefficiens* is unable to grow with trehalose as the sole carbon source (41). Trehalose can also be bound covalently to other molecules. In mycobacteria and corynebacteria, it forms glycolipids (cord factors) which are an integral part of the cell wall (62). Well documented is the role of trehalose as a chemical chaperone that protects cells and higher organisms from adverse conditions, such as osmotic stress, heat, or oxidative stress (62). Moreover, trehalose and its precursor T6P can act as signaling molecules for metabolic or developmental processes in yeast and plants (63–65). Notably, trehalose also plays a role in various bacterium-plant interactions either as a stress protectant or virulence factor. For example, trehalose protects Xanthomonas citri subsp. *citri* from oxidative and osmotic stress, and *otsA* mutants show reduced leaf colonization and pathogenicity on oranges (66). Likewise, Pseudomonas syringae pv. tomato mutants unable to synthesize trehalose are compromised in osmostress tolerance and phyllosphere fitness, while analogous mutants of Pseudomonas aeruginosa PA14 exhibit reduced pathogenicity on Arabidopsis thaliana leaves (67, 68). Yet another example is the plant pathogen Ralstonia solanacearum which encodes two functional TPS enzymes. Mutants lacking the OtsA homologue are more sensitive to osmotic stress, less virulent, and lack competitiveness (69). Most remarkably, the second TPS homologue is an effector protein which is translocated into plant cells via a type III secretion system where it triggers a hypersensitive response when recognized by the plant immune system (70).

What is the function of trehalose in the interaction of *B. diazoefficiens* with its soybean host plant? Our previous findings (50) combined with the results reported here imply that during early stages of host infection, *B. diazoefficiens* cells are stress exposed and induce trehalose synthesis via the T6P pathway. We postulate that trehalose serves as an intracellular osmoprotectant in those cells because of the following. (i) The sensitivity of Δ(*otsCB-otsA*) mutant cells to ionic (NaCl, MgCl_2_, MgSO_4_) and nonionic (sorbitol) osmostress (but not oxidative or alkaline pH stress) was greatly increased relative to wild-type cells. (ii) Exogenous trehalose added to the agar medium restored tolerance of cells to sorbitol-induced stress. (iii) Osmotolerance and symbiotic efficiency were both restored in recombinant Δ(*otsCB-otsA*) mutant cells synthesizing the well-characterized chemical chaperones ectoine and hydroxyectoine (26). Indeed, symbiotic defects of trehalose biosynthesis mutants could also be restored by addition of trehalose to the plant growth medium but only if the annotated MFS family sugar transporter OtsC was present. Conversely, a wild-type strain producing the intracellular E. coli trehalase TreF could not be rescued by addition of exogenous trehalose, further corroborating the need for intracellular accumulation of trehalose as a stress protectant in early symbiosis.

The impaired symbiotic competitiveness of the *otsBA*-complemented Δ*ecfG* mutant compared to that of the complemented Δ(*otsCB-otsA*) mutant indicates that trehalose biosynthesis via the T6P pathway is not the only GSR-controlled process contributing to symbiotic fitness. Whether tolerance to elevated pH, which is GSR dependent but independent of the T6P pathway (Fig. S3D), is symbiosis relevant remains to be elucidated. Evidently, competition for nodule occupancy is not only related to the infection thread stage but also involves numerous other steps, such as adaptation to the plant growth substrate (or the soil under field conditions) and nutrient availability, root colonization and attachment, release into plant cells, and intracellular proliferation. It is plausible that GSR-controlled functions other than trehalose synthesis play a role in one or more of these processes.

Information about the local physicochemical conditions prevailing in microcolonies and infection threads is scarce, making it difficult to speculate about the nature of the stress signal(s) that triggers the GSR-controlled trehalose synthesis in infecting *B. diazoefficiens* cells. A cellular consequence common to ionic and nonionic hyperosmotic stress is water efflux, which may activate the GSR and is in line with the elevated desiccation sensitivity of GSR-deficient *B. diazoefficiens* mutants (49). In any case, early infection stages impose osmotically stressful conditions on *B. diazoefficiens* cells, which they ameliorate by the synthesis of the chemical chaperone trehalose. The decline in GSR activity at later symbiotic stages indicates that bacteria present inside symbiosomes experience more favorable conditions that no longer require protective chaperones. In this context, it is notable that *B. diazoefficiens* is among the most salt-sensitive rhizobia, which may correlate with its need for chaperones during infection, whereas GSR- and trehalose-deficient mutants of the naturally more salt-tolerant species Sinorhizobium meliloti and Rhizobium leguminosarum bv. trifolii, respectively, are symbiotically proficient (40, 71, 72). Nonetheless, S. meliloti also induces the GSR during early symbiosis (15, 72), and trehalose biosynthesis mutants of both S. meliloti and R. leguminosarum bv. trifolii lack competitiveness for nodulation (40, 47). We thus conclude that rhizobia generally experience adverse osmotic conditions during early infection of their hosts, and trehalose accumulation is a common response to overcome the infection stress and ensure competitiveness. The pronounced sensitivity of *B. diazoefficiens* renders this species particularly dependent on osmoprotective mechanisms. Accordingly, enhanced trehalose synthesis is particularly crucial for symbiotic fitness of *B. diazoefficiens*, as it appears to be the sole resistance mechanism to overcome the stress during early infection, whereas other rhizobial species may rely on alternative protective means or are intrinsically more stress tolerant.

## MATERIALS AND METHODS

### Bacterial strains and cultivation.

Strains and plasmids used in this study are listed in Table S1A in the supplemental material. E. coli was grown in LB medium (73) at 37°C. Antibiotics were added where appropriate at the following concentrations (in micrograms per milliliter): ampicillin (agar plates, 200, liquid cultures, 100), kanamycin (30), streptomycin (50), and tetracycline (10). *B. diazoefficiens* was routinely grown at 30°C in PSY medium (74) supplemented with 0.1% l-(+)-arabinose. Antibiotics were added where appropriate as follows (in micrograms per milliliter): chloramphenicol (20, for counterselection of E. coli), kanamycin (100), spectinomycin (100), streptomycin (50), and tetracycline (agar plates, 50; liquid cultures, 25). To induce carbon starvation, morpholinepropanesulfonic acid (MOPS)-buffered minimal medium (75) or V3 minimal medium (76) lacking any carbon source was used.

10.1128/mBio.00390-21.10 TABLE S1 (A) Strains and plasmids used in this study. (B) Oligonucleotides used in this study. Download Table S1, DOCX file, 0.04 MB. Copyright © 2021 Ledermann et al. 2021 Ledermann et al. https://creativecommons.org/licenses/by/4.0/ This content is distributed under the terms of the Creative Commons Attribution 4.0 International license.

### Plasmid and strain constructions.

For deletion mutant generation, the pREDSIX system was used as described previously (77). In short, flanking regions (650- to 900-bp up- and downstream DNA of the region to be deleted) were PCR amplified (see Table S1B for primers and oligonucleotides used in this study) and cloned in tandem orientation into vector pREDSIX. These plasmids were linearized at the unique site between the two flanking regions and an antibiotic resistance cassette excised from pRGD derivatives was inserted. Final plasmids were conjugated into *B. diazoefficiens* recipient strains by biparental matings using E. coli strain S17-1 λ*pir* as the donor. Clones that underwent double crossovers were selected by their nonfluorescent phenotype and double checked by PCR using primers binding to the antibiotic resistance cassette (oriented outwards) in combination with primers binding to the genomic region outside the flanking regions used for homologous recombination (oriented toward the deleted region) (see Table S1B). For markerless deletions, a similar method was used, and isolation and verification was done as described (77). For detailed cloning and mutagenesis procedures, see Text S1.

### RNA work and reverse transcription-PCR (RT-PCR).

RNA was extracted from 12 to 20 ml of bacterial culture (optical density at 600 nm [OD_600_] = 0 .3 to 0.6). Cells were harvested by adding 10% (vol/vol) stop solution (5% [vol/vol] Tris-HCl-buffered phenol [pH 5] [Applichem]) and subsequent centrifugation (5,000 × *g*, 10 min, 4°C). Pellets were resuspended in 600 μl TRI reagent (Zymo Research Corp.) and 300 μl of 0.1 mm zirconia beads (Biospec Products, Inc.) were added. Cells were lysed by vigorous shaking (three times, 30 s each time) using a CapMix apparatus (3M ESPE). Beads were removed by centrifugation (15,000 × *g*, 1 min, 4°C), and RNA was extracted from supernatant using the Direct-zol RNA MiniPrep kit (Zymo Research Corp.) according to the manufacturer’s recommendations. After RNA elution in 85 μl water, 1 μl of RNasin Plus RNase inhibitor (Promega AG) was added. Remaining genomic DNA was removed using the RapidOut DNA removal kit (ThermoFisher Scientific) according to the manufacturer’s protocol. Synthesis of cDNA was performed using the Omniscript reverse transcription kit (Qiagen AG) and random hexameric primers (Invitrogen) following the manufacturer’s instructions. cDNA was used for PCR analysis of the *otsCB-otsA* transcriptional organization using primer pairs RT-1/-3, RT-1/-4, and RT-5/-7.

### Metabolite measurements.

For determination of trehalose content in different strains of *B. diazoefficiens*, cells were grown to exponential phase in PSY medium. Cells were harvested by centrifugation (5,000 × *g*, 10 min, 20°C), washed in V3 minimal medium without a carbon source and inoculated in either PSY medium or V3 minimal medium without a carbon source and cultivated at 30°C overnight. Harvesting and metabolite extraction were done according to a modified version of a previously described protocol (61). Briefly, 2 ml of culture was harvested in prechilled tubes by fast centrifugation (21,000 × *g*, 30 s, 0°C), and pellets were immediately frozen in liquid nitrogen. Cell pellets were stored at −80°C until extraction. For extraction, 150 μl of −20°C 60% (vol/vol) methanol in water was added per OD_600_ unit. Samples were resuspended by vortexing and incubated for 20 min at −20°C, including three rounds of vigorous vortexing. Samples were centrifuged (16,100 × *g*, 5 min, 0°C), and supernatants were transferred to new, prechilled tubes and stored at −80°C until analysis. Mass spectrometric measurement of metabolites was done as described previously (78).

For specific determination of trehalose, glycine betaine, hydroxyectoine, and ectoine in strains 110*spc*4, 9871, 9987, and 71-87, cells were grown to mid-exponential phase in 10 ml PSY medium and subsequently salt stressed by addition of 5 M NaCl stock solution to a final concentration of 40 mM followed by incubation for another 5 h. Cells were harvested by centrifugation (3,220 × *g*, 10 min, 4°C), washed twice in ice-cold 0.9% NaCl solution, and washed again in ice-cold milliQ water. Pellets were flash-frozen in liquid nitrogen and stored at −80°C until metabolite extraction. Metabolites were extracted in 300 μl of −20°C 60% (vol/vol) methanol in water for 20 min at −20°C with repeated vortexing.

Quantitative liquid chromatography-mass spectrometry (LC-MS) was performed according to a modified version of a previously described protocol (79, 80) using Thermo Scientific Ultimate 3000 ultrahigh-performance liquid chromatograph (UHPLC) coupled to a Q Exactive plus mass spectrometer (ThermoFisher Scientific) with a heated ESI source. For LC separation, a Waters Acquity ethylene bridged hybrid (BEH) amide column (100 × 2.1 mm; pore size, 1.7 μm) was used, and MS analysis was performed in both Fourier transform MS (FTMS) modes at mass resolution of 35,000 (at *m/z* 200). Source parameters were set as follows: vaporizer temperature, 400°C; sheath gas, 50; auxiliary gas, 20; S-lens radio frequency (RF) level, 50.0; capillary temperature, 275°C. Prior to analysis, 5 μl of the metabolite-containing supernatant was mixed with 45 μl LC mobile phase at initial conditions. (Hydroxy)ectoine and glycine betaine were measured in positive FTMS mode with the source voltage set at 3.5 kV and a scan range of *m/z* 85 to 600. Trehalose was measured in negative FTMS mode with the source voltage set at 3.0 kV and a scan range of *m/z* 100 to 800. Relative metabolite concentrations (in arbitrary units) were calculated from peak area normalized to total ion chromatogram and OD_600_ of the extracted cell pellet.

### Plant tests.

Soybean seeds [Glycine max (L.) Merr.] cv. Green Butterbeans (Johnny’s Selected Seeds, Albion, ME, USA) were sterilized and germinated as described previously (53). Inoculation, plant growth conditions, and nitrogenase assay have been described previously (81). When exogenous sugars were tested, the 100-ml volume of Jensen medium, normally added to the vermiculite-filled 180-ml jars before autoclaving, was reduced to 80 ml of 1.25-fold concentrated medium. Twenty milliliters of filter-sterilized 0.5 M sugar solutions (corresponding to 10 mmol of sugar) was added to the jars just before planting the seedlings. Control plants received the same volume of sterile water. Counting of infection threads was done as described previously (53).

10.1128/mBio.00390-21.1 TEXT S1 Supplemental methods. Download Text S1, DOCX file, 0.04 MB. Copyright © 2021 Ledermann et al. 2021 Ledermann et al. https://creativecommons.org/licenses/by/4.0/ This content is distributed under the terms of the Creative Commons Attribution 4.0 International license.
