# Supplementary material for: Bradyrhizobium diazoefficiens Requires Chemical Chaperones To Cope with Osmotic Stress during Soybean Infection
Source: mBio. 2021 Mar 30;12(2):e00390-21. doi: 10.1128/mBio.00390-21 (PMC8092242; doi:10.1128/mBio.00390-21)
Supplement: FIG S1 [file mBio.00390-21-sf001.pdf]

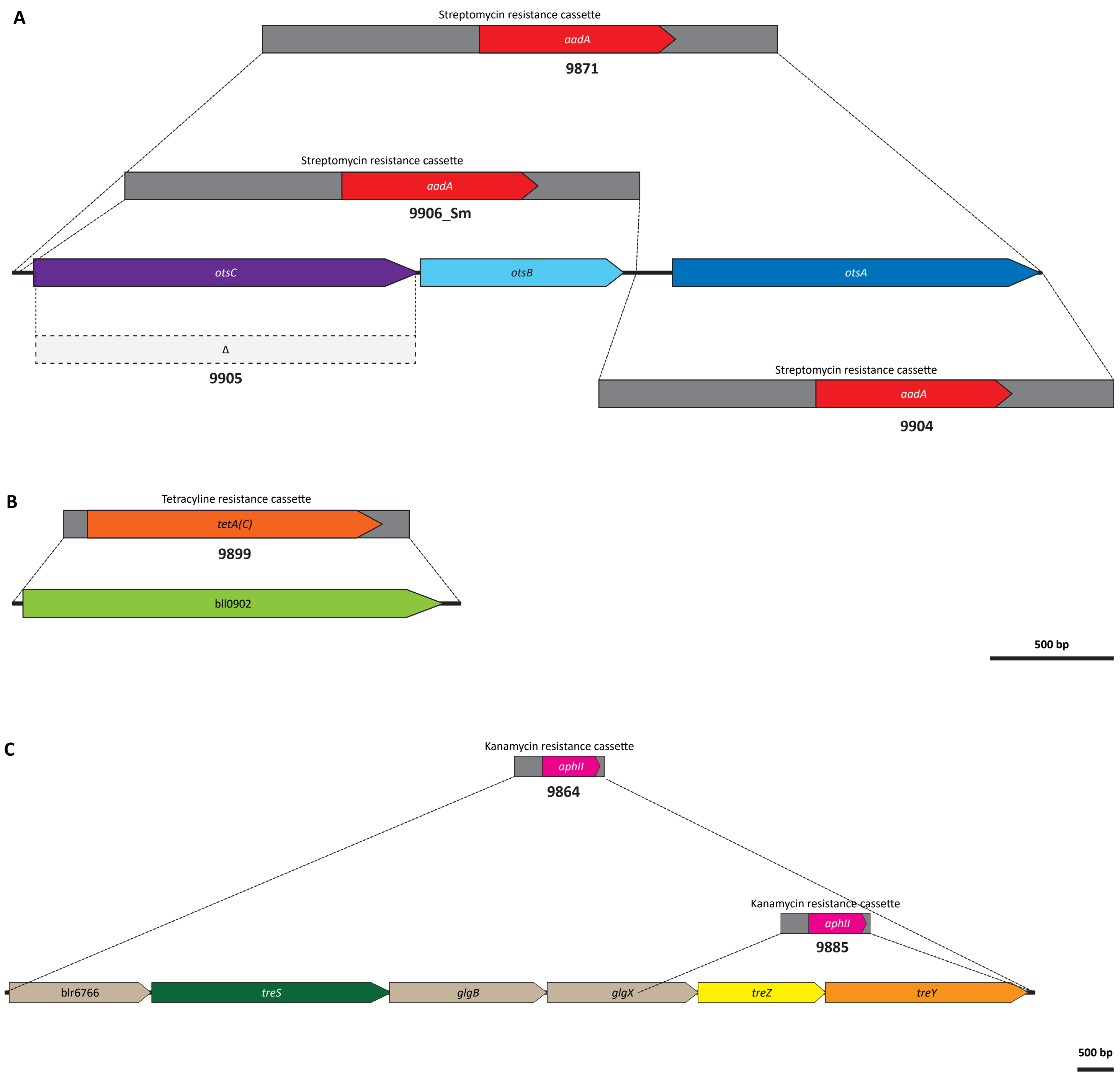

FIG. S1. Genetic map of three *B. diazoefficiens* loci encoding trehalose biosynthesis genes and genotype of respective deletion mutants. Mutant strain numbers are indicated below the deleted regions or the inserted antibiotic resistance gene cassettes. Locus encoding genes for trehalose biosynthesis via the T6P pathway (A): *otsA* (bll0322) encoding trehalose-6-phosphate synthase (dark blue), *otsB* (bll0323) encoding trehalose-6-phosphate phosphatase (light blue), *otsC* (bll0324) encoding an MFS-like sugar transport protein which putatively mediates trehalose uptake (purple). Constructed mutants lack either the entire locus (Δ(*otsCB-otsA*)::*aadA*; strain 9871), *otsA* (Δ*otsA::aadA*; 9904), *otsC* and *otsB* (Δ*otsCB::aadA*; 9906_Sm), or *otsC* (Δ*otsC* markerless in-frame deletion; 9905). The bll0902 (light green) locus encoding a putative trehalose synthase which converts maltose into trehalose (B). In mutant strain 9899 (Δbll0902::*tetA*(C)), bll0902 is deleted. Locus encoding the putative operon for trehalose biosynthesis genes via the TreS and the TreZY pathways (C): *treS* (blr6767; dark green), *treZ* (blr6771; yellow), *treY* (blr6770; orange) coding for trehalose synthase, malto-oligosyl trehalose trehalohydrolase and malto oligosyl trehalose synthase, respectively, and three genes related to glycogen metabolism: blr6766 (putative glucanohydrolase) *glgB* (blr6768; putative 1,4 α-glucan branching enzyme) and *glgX* (blr6769; putative glycogen debranching enzyme). Generated mutants carry deletions of the entire gene cluster (Δ(blr6766-*treS-glgB-glgX-treZ-treY)*::aphII; strain 9864) or *treZY* and the 3' end of *glgX* (Δ(*glgX'-treZY*)::aphII; 9885). Maps are drawn to scale with the upper scale bar applying to panels A and B and the lower to panel C.

Ledermann et al., Fig. S1
