# Supplementary material for: Bradyrhizobium diazoefficiens Requires Chemical Chaperones To Cope with Osmotic Stress during Soybean Infection
Source: mBio. 2021 Mar 30;12(2):e00390-21. doi: 10.1128/mBio.00390-21 (PMC8092242; doi:10.1128/mBio.00390-21)
Supplement: FIG S2 [file mBio.00390-21-sf002.pdf]

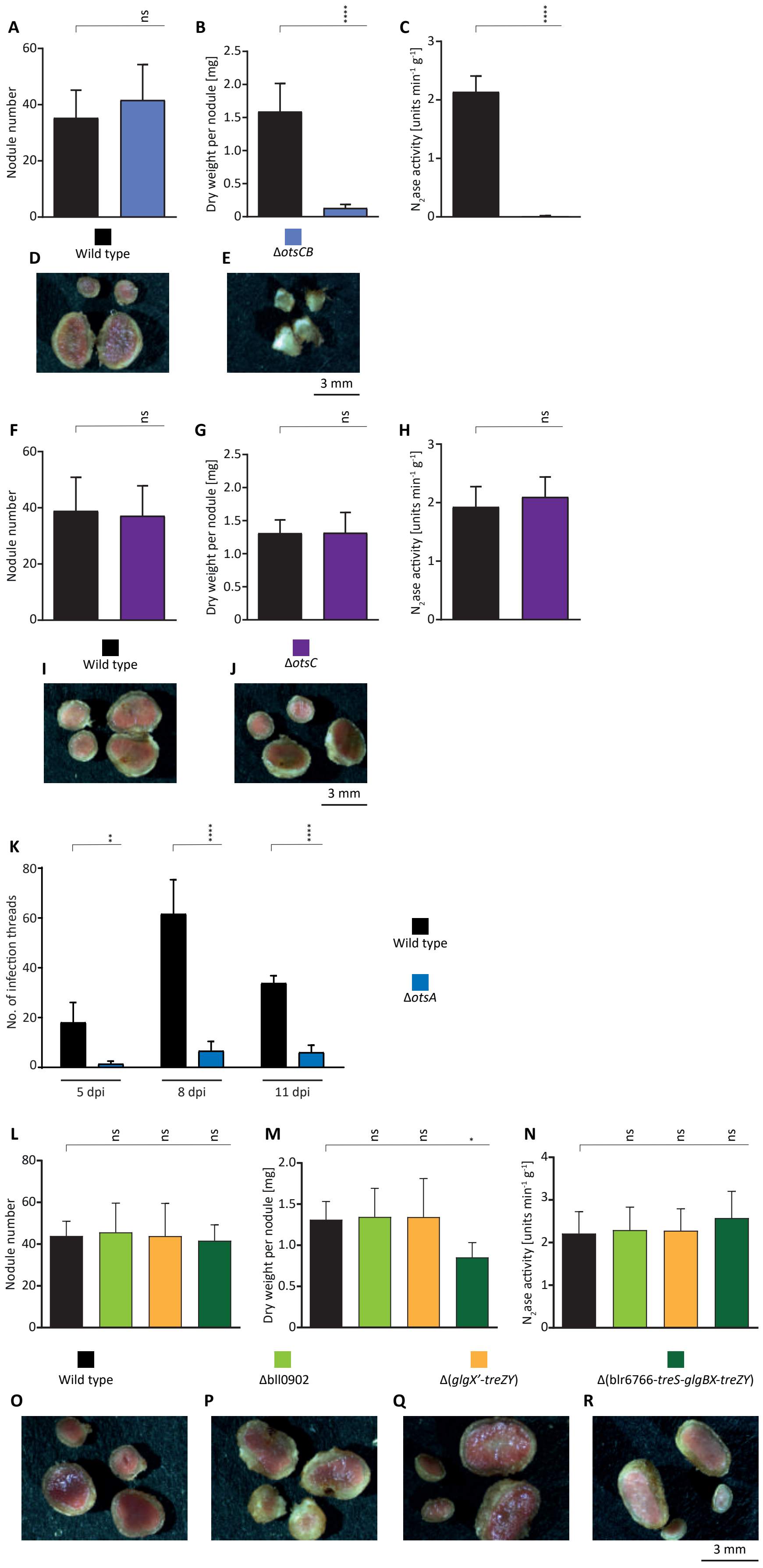

Ledermann et al., Fig. S2

FIG. S2. Symbiotic phenotype of additional mutants in trehalose biosynthesis and related genes. Cells of *B. diazoefficiens* wild type (strain 110*spc*4), Δ*otsCB* (9906_Sm), Δ*otsC* (9905), Δbll0902 (9899), Δ(*glgX'-treZY)* (9985), and Δ(blr6766-*treS-glgBX-treZY*);(9964) were inoculated on soybean seedlings and harvested 21 dpi (n≥9). Plants were evaluated for nodule number (A, F, and L), dry weight per nodule (B, G, and M), and nitrogenase activity measured by acetylene reduction (C, H, and N). Cross sections of representative nodules showing overall nodule morphology and presence of reddish colour indicative for leghemoglobin (D, E, I, J, O, P, Q, and R). Number of infection threads formed by a GusA-tagged Δ*otsA* mutant compared to a GusA-tagged wild type (GusA-1) at 5, 8, and 11 dpi (K). Displayed are means and error bars represent SD (n=5). Statistical significances of pairwise comparisons made between columns marked with a vertical tick and adjacent columns under horizontal lines were determined using a two-tailed Student's t test (A, B, C, F, G, and H) or one-way ANOVA with Šidák multiple comparison correction (K, L, M, and N); ns P≥0.05, * P≤0.05, ** P≤0.01, **** P≤0.0001.
