# Supplementary material for: Bradyrhizobium diazoefficiens Requires Chemical Chaperones To Cope with Osmotic Stress during Soybean Infection
Source: mBio. 2021 Mar 30;12(2):e00390-21. doi: 10.1128/mBio.00390-21 (PMC8092242; doi:10.1128/mBio.00390-21)
Supplement: FIG S3 [file mBio.00390-21-sf003.pdf]

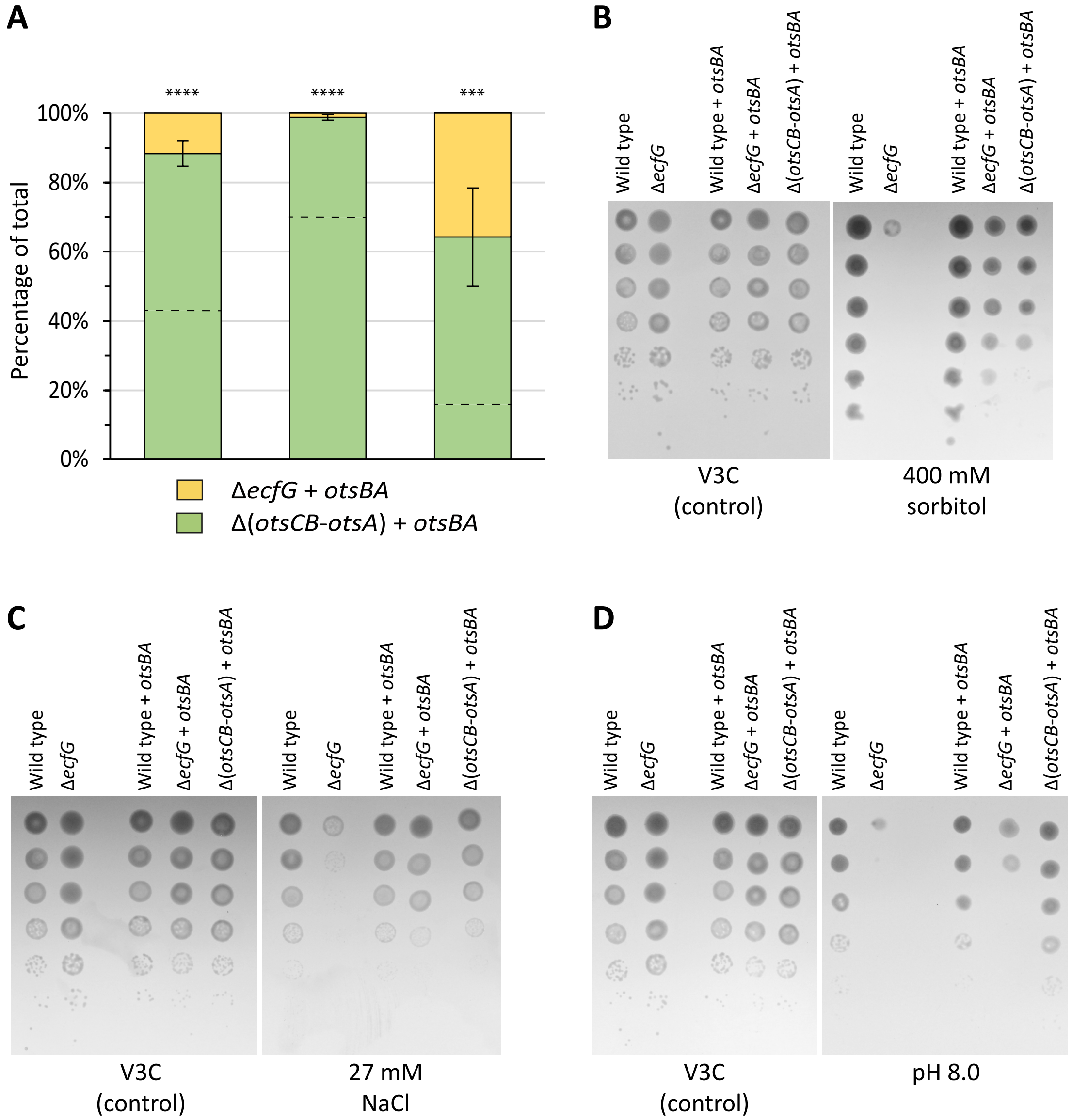

FIG. S3. Trehalose synthesis via the T6P pathway cannot fully restore symbiotic competitiveness and stress tolerance of the Δ*ecfG* mutant. (A) Mixtures of the *B. diazoefficiens* Δ(*otsCB-otsA*) and Δ*ecfG* mutant strains, both complemented with constitutively expressed *otsBA* genes (strain 71-1687 and 8404-1687, respectively), containing a total of approx. 15'000 cells at three different ratios (71-1687 : 8404-1687 = 43% : 57% (left stacked bar), 70% : 30% (middle), 16% : 84% (right); dashed horizontal lines) were inoculated on soybean seedlings. The nodules of six individual plants per inoculum were harvested 19 dpi and crushed with a plastic pistil in a 2-ml Eppendorf tube. Aliquots of serial dilutions of the resulting 18 extracts were plated in parallel on PSY agar plates selective for one of the two strains. Colony-forming units (CFUs) were counted and the relative abundance of both strains in the nodules of the six individual plants was calculated. Significance of differences detected by one-sample Student's t tests between ratios in the inocula and in reisolated bacteroids is indicated above the stacked bars. *** P ≤0.001, **** P ≤0.0001.
Cells of *B. diazoefficiens* wild type (strain 110*spc*4), ΔecfG; (8404), and strains harboring constitutively expressed *otsBA* genes in the wild-type (1687), Δ*ecfG* (8404-1687), and Δ(*otsCB-otsA*) (71-1687) background were grown to mid-exponential phase in V3C medium, adjusted to an OD600=0.1, and 4-µl aliquots of serial dilutions were spotted on V3C agar plates imposing either osmostress (B), salt stress (C), or alkaline pH stress (D).
