# Supplementary material for: Bradyrhizobium diazoefficiens Requires Chemical Chaperones To Cope with Osmotic Stress during Soybean Infection
Source: mBio. 2021 Mar 30;12(2):e00390-21. doi: 10.1128/mBio.00390-21 (PMC8092242; doi:10.1128/mBio.00390-21)
Supplement: FIG S4 [file mBio.00390-21-sf004.pdf]

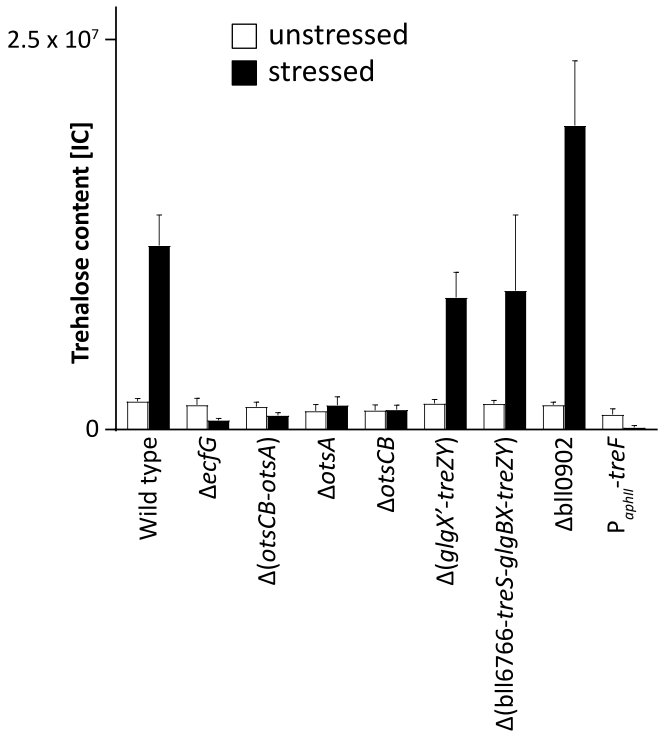

Comparison strain/condition 1 *vs.* strain/condition 2		Significance
**1**	**2**	
wild type unstressed	Δ*ecfG* unstressed	ns
wild type unstressed	Δ(*otsBC-otsA*) unstressed	ns
wild type unstressed	Δ*otsA* unstressed	ns
wild type unstressed	Δ*otsBC* unstressed	ns
wild type unstressed	Δ(*glgX'-treZY*) unstressed	ns
wild type unstressed	Δ(bll6766-*treS-glgBX-treZY*) unstressed	ns
wild type unstressed	Δbll0902 unstressed	ns
wild type unstressed	P_{aphII}-*treF* unstressed	ns
wild type stressed	Δ*ecfG* stressed	****
wild type stressed	Δ(*otsBC-otsA*) stressed	****
wild type stressed	Δ*otsA* stressed	****
wild type stressed	Δ*otsBC* stressed	****
wild type stressed	Δ(*glgX'-treZY*) stressed	*
wild type stressed	Δ(bll6766-*treS-glgBX-treZY*) stressed	ns
wild type stressed	Δbll0902nstressed	****
wild type stressed	P_{aphII}-*treF* stressed	****
wild type unstressed	wild type stressed	****
Δ*ecfG* unstressed	Δ*ecfG* stressed	ns
Δ(*otsBC-otsA*) unstressed	Δ(*otsBC-otsA*) stressed	ns
Δ*otsA* unstressed	Δ*otsA* stressed	ns
Δ*otsBC* unstressed	Δ*otsBC* stressed	ns
Δ(*glgX'-treZY*) unstressed	Δ(*glgX'-treZY*) stressed	****
Δ(bll6766-*treS-glgBX-treZY*) unstressed	Δ(bll6766-*treS-glgBX-treZY*) stressed	****
Δbll0902 unstressed	Δbll0902 stressed	****
P_{aphII}-*treF* unstressed	P_{aphII}-*treF* stressed	ns

FIG. S4. Trehalose content of *B. diazoefficiens* wild-type and mutant cells grown under stressed and unstressed conditions. Cells of the wild type (strain 110*spc*4*),* or Δ*ecfG* mutant (8404), Δ(*otsCB-otsA*) (9871), Δ*otsA* (9904), Δ*otsCB* (9906_Sm), Δ(*glgX'-treZY*) (9985), Δ(blr6766-*treS-glgBX-treZY*) (9964), Δbll0902 (9899), and of a wild type-derived strain expressing *E. coli treF* from a constitutive promoter (TreF-1) were grown in PSY medium and transferred either to fresh PSY (unstressed) or V3 minimal medium lacking a carbon source (stressed) and incubated for a further 12 h. Cells were harvested, washed, extracted, and trehalose content was measured as described in Materials and Methods. Relative trehalose content is indicated as ion count (IC). n=6, displayed are means and error bars represent SD. Statistical significances of pairwise comparisons listed in the table below the graph were determined using one-way ANOVA with Šidák multiple comparison correction; ns P≥0.05, * P≤0.05, **** P≤0.0001.
