# Supplementary material for: Bradyrhizobium diazoefficiens Requires Chemical Chaperones To Cope with Osmotic Stress during Soybean Infection
Source: mBio. 2021 Mar 30;12(2):e00390-21. doi: 10.1128/mBio.00390-21 (PMC8092242; doi:10.1128/mBio.00390-21)
Supplement: FIG S5 [file mBio.00390-21-sf005.pdf]

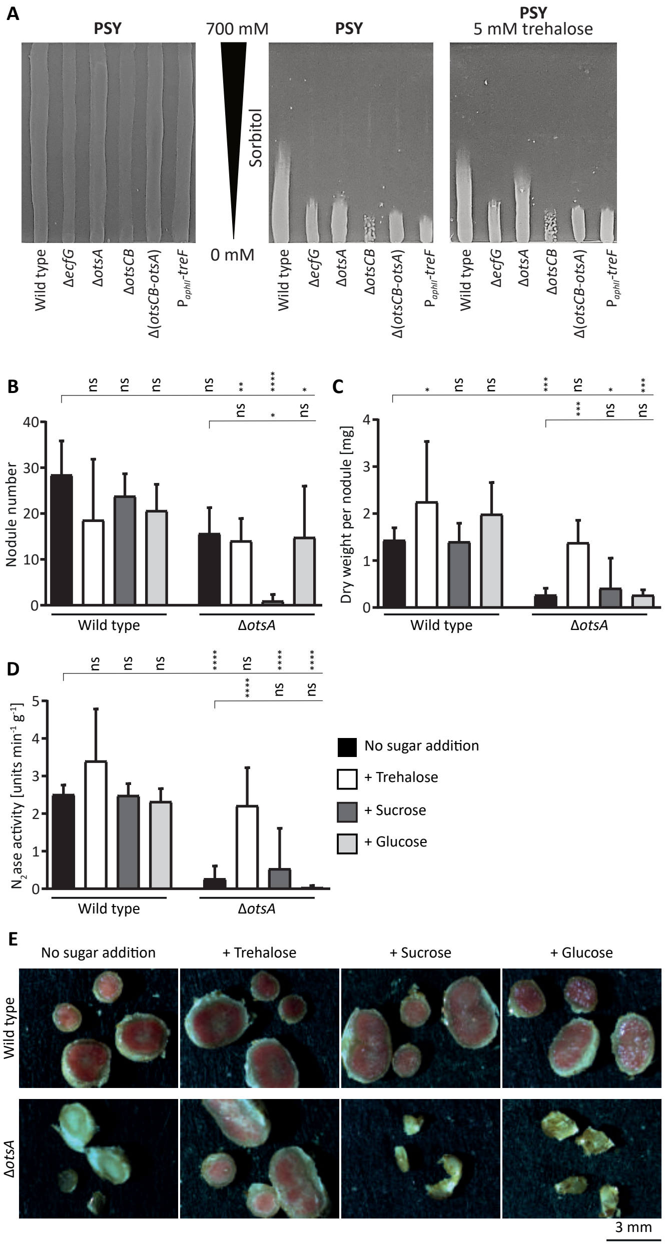

FIG. S5. Exogenous trehalose can rescue a Δ*otsA* mutant under hyperosmotic conditions and in symbiosis. *B. diazoefficiens* wild type (strain 110*spc*4), Δ*ecfG* (8404), Δ(*otsCB-otsA*) (9871), Δ*otsA* (9904), Δ*otsCB* (9906_Sm), and a strain expressing *E. coli treF* from a constitutive promoter in a wild-type background (treF-1) were adjusted to OD600=0.01. Strains then were streaked on PSY agar plates containing a sorbitol gradient ranging from 0 mM to 700 mM and supplemented without (center) or with 5 mM trehalose (right). No sorbitol was added to the control plate (left) (A). Reduced growth of the Δ*otsCB* mutant likely is due to increased trehalose-6-phosphate levels resulting in sugar phosphate toxicity (see (5) and references therein). For evaluating symbiotic phenotypes, wild-type (strain 110*spc*4) or Δ*otsA* mutant (9904) cells were inoculated on soybean seedlings. These were grown in 180-ml jars filled with vermiculite which was soaked with mineral salts solution and supplemented with 10 mmoles of the indicated sugar or no additional sugar (for details, see Materials and Methods). Plants were harvested 21 dpi and evaluated for nodule number (B), dry weight per nodule (C), and nitrogenase activity measured by acetylene reduction (D). Cross sections of representative nodules showing overall nodule morphology and presence of reddish color indicative for leghemoglobin (E). Displayed are means and error bars represent SD (n≥8). Statistical significances of pairwise comparisons made between columns marked with a vertical tick and adjacent columns under horizontal lines were determined using one-way ANOVA with Šidák multiple comparison correction; ns P≥0.05, * P≤0.05, ** P≤0.01, *** P≤0.001, **** P≤0.0001. Note that the data shown in panels B, C, and D, and E of this figure were generated in the same experiment underlying the data set shown in Fig. 3. Hence, the same data are shown for the reference conditions (wild type and Δ*otsA* mutant, with and without trehalose) in both figures. Statistical analysis was performed on the entire data set.
