# Supplementary material for: Bradyrhizobium diazoefficiens Requires Chemical Chaperones To Cope with Osmotic Stress during Soybean Infection
Source: mBio. 2021 Mar 30;12(2):e00390-21. doi: 10.1128/mBio.00390-21 (PMC8092242; doi:10.1128/mBio.00390-21)
Supplement: FIG S6 [file mBio.00390-21-sf006.pdf]

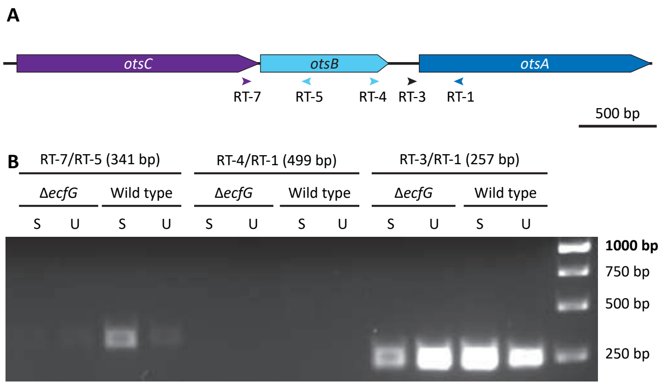

FIG. S6. Transcriptional organisation and regulation of the *B. diazoefficiens otsCB-otsA* gene region. Cells of the wild type (strain 110*spc*4) and Δ*ecfG* mutant (8404) were grown in PSY medium (unstressed, U) or PSY medium containing 30 mM NaCl (stressed, S). Total RNA was extracted and cDNA synthesized using random hexameric primers. Genetic organization and primer binding sites (A) used for endpoint RT-PCR resulting in the amplification products shown in (B). *otsC* and *otsB* form an operon while *otsA* is transcribed separately. Unlike *otsCB*, transcription of *otsA* is only partially dependent on σ^{EcfG}.

Ledermann et al., Fig. S6
