# Supplementary material for: Bradyrhizobium diazoefficiens Requires Chemical Chaperones To Cope with Osmotic Stress during Soybean Infection
Source: mBio. 2021 Mar 30;12(2):e00390-21. doi: 10.1128/mBio.00390-21 (PMC8092242; doi:10.1128/mBio.00390-21)
Supplement: FIG S7 [file mBio.00390-21-sf007.pdf]

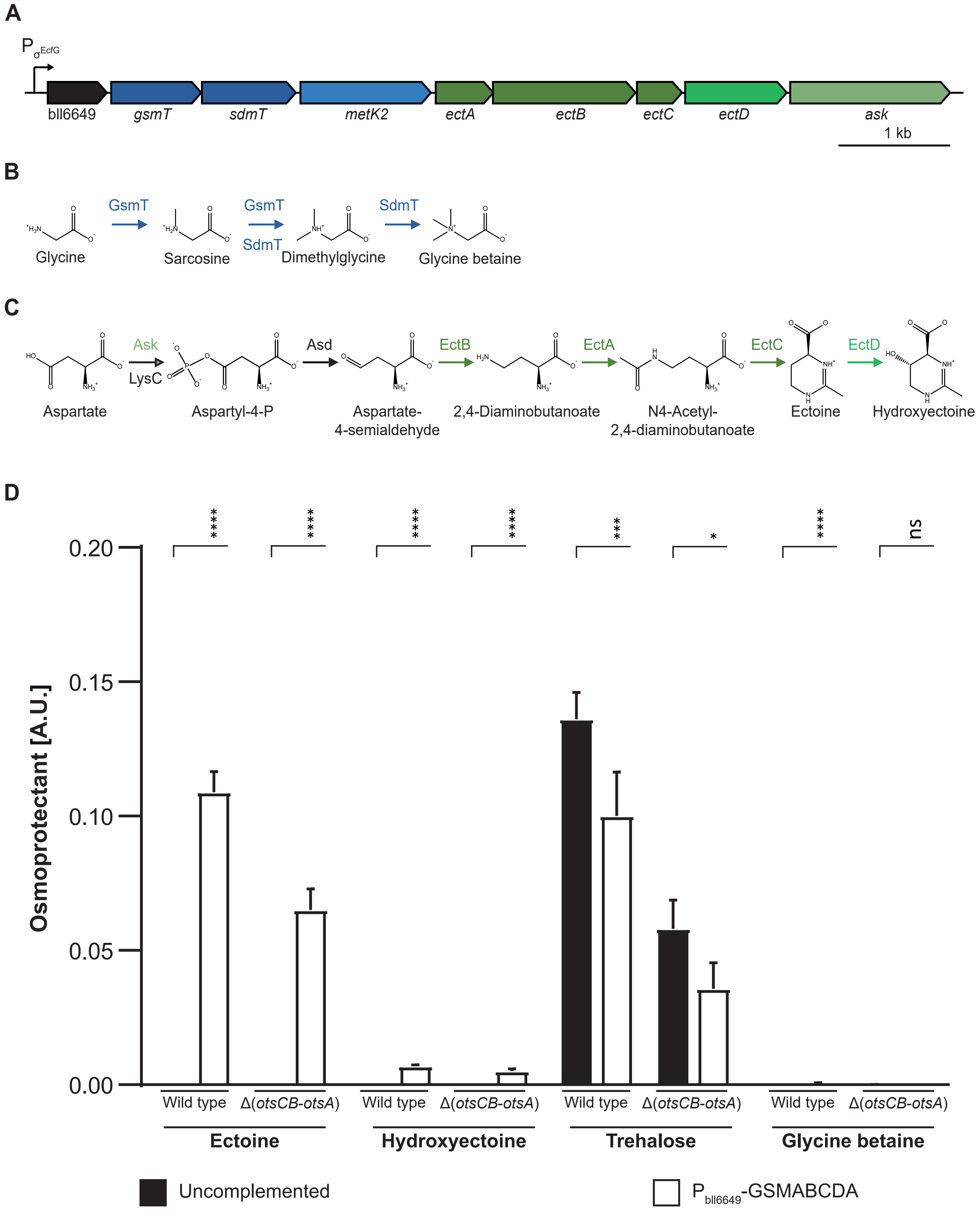

Ledermann et al., Fig. S7 (continued)

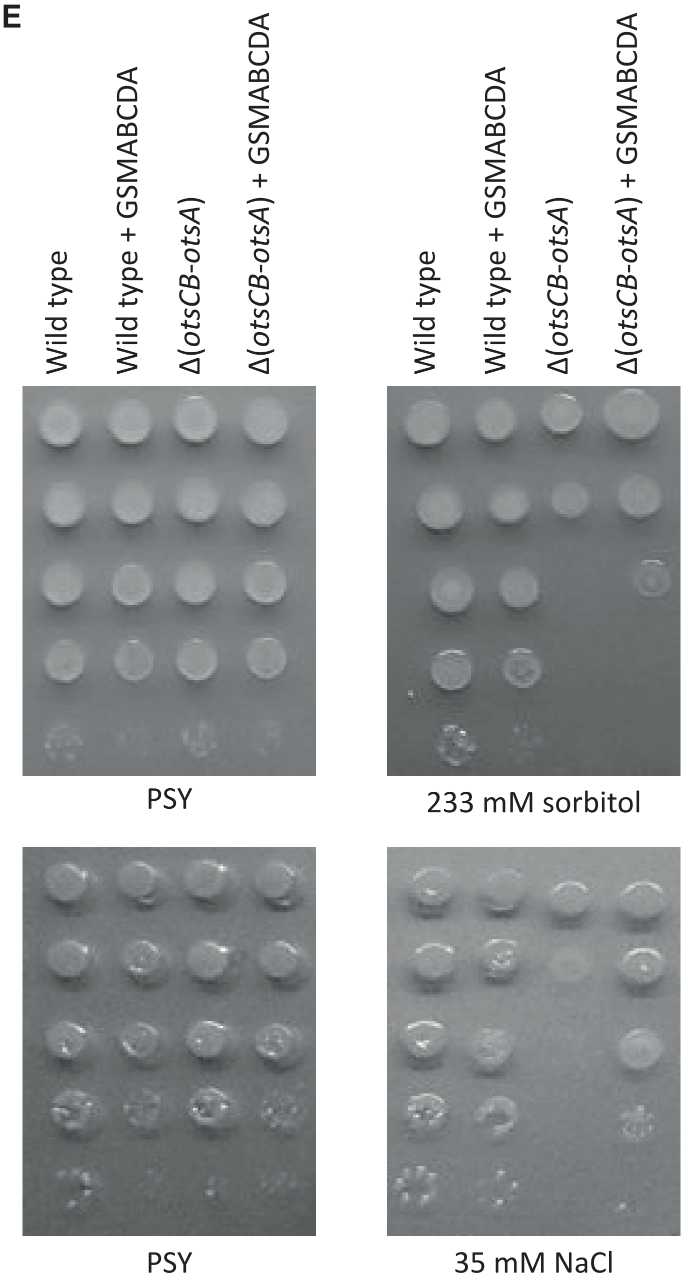

FIG. S7. Complementation of trehalose biosynthesis mutants by expression of recombinant genes encoding biosynthesis for different chemical chaperones. The chaperone biosynthesis genes were integrated as an artificial operon downstream of the σ^{EcfG}-controlled gene bll6649. Genes for glycine betaine biosynthesis (*gsmT* and *sdmT*; dark blue) and the methyl donor (SAM)-regenerating enzyme MetK2 (light blue) were derived from *Halorhodospira halochloris.* Genes for ectoine (*ectABC*; dark green), hydroxyectoine (*ectD;* bright green) and a feedback-insensitive aspartate kinase (*ask*; light green) were derived from *Pseudomonas stutzeri.* Both gene clusters including the accessory function genes *metK2* and *ask* are present in the same order in the respective host organism (A). Biosynthesis of glycine betaine. GsmT (glycine/sarcosine methyltransferase) and SdmT (sarcosine/dimethylglycine methyltransferase) successively N methylate glycine using SAM as a methyl donor. Notably, this pathway differs from many other glycine betaine-producing organisms, which rather employ oxidation of choline (B). Biosynthesis of ectoine and hydroxyectoine. Biosynthesis starts from aspartate-4-semialdehyde which is aminated to 2,4-diaminobutanoate by EctB using glutamate as an aminodonor. EctA transfers the acetyl group from acetyl-CoA to the γ-amino group forming N4-acetyl-2,4-diaminobutanoate which in turn is cyclized to ectoine by EctC. Hydroxylation of ectoine to hydroxyectoine by EctD employs molecular oxygen, and α-ketoglutarate as an additional electron donor. Biosynthesis of aspartate-4-semialdehyde, which is also an intermediate in homoserine and lysine biosynthesis, involves the endogenous enzymes LysC and Asd (black) (C). Cells of wild type (strain 110*spc*4), Δ(*otsCB-otsA*) (9871), and the respective backgrounds expressing the heterologous biosynthesis operon *gsmT-sdmT-metK2-ectABCD-ask* (GSMABCDA; strains 9987 and 71-87) were grown to mid-exponential phase in PSY before the σ^{EcfG}-dependent bll6649 promoter located upstream of the operon was induced by addition of 40 mM NaCl. After further incubation for 5 h, cell extracts were prepared and chemical chaperones were determined by LC-MS measurements as described in Materials and Methods (D). Displayed are means and error bars represent SD (n=5). Statistical significances of pairwise comparisons made between columns marked with a vertical tick and adjacent columns under horizontal lines were determined for each individual compound using one-way ANOVA with Šidák multiple comparison correction; ns $P \geq 0.05$, * $P \leq 0.05$, *** $P \leq 0.001$, **** $P \leq 0.0001$. Suspensions of the indicated strains grown identically as described for (D) were adjusted to an OD600=0.1, serially diluted and then 4 µl aliquots were spotted on PSY agar plates with the indicated ionic (NaCl) and non-ionic (sorbitol) osmostress (E).
