# Supplementary material for: Bradyrhizobium diazoefficiens Requires Chemical Chaperones To Cope with Osmotic Stress during Soybean Infection
Source: mBio. 2021 Mar 30;12(2):e00390-21. doi: 10.1128/mBio.00390-21 (PMC8092242; doi:10.1128/mBio.00390-21)
Supplement: FIG S8 [file mBio.00390-21-sf008.pdf]

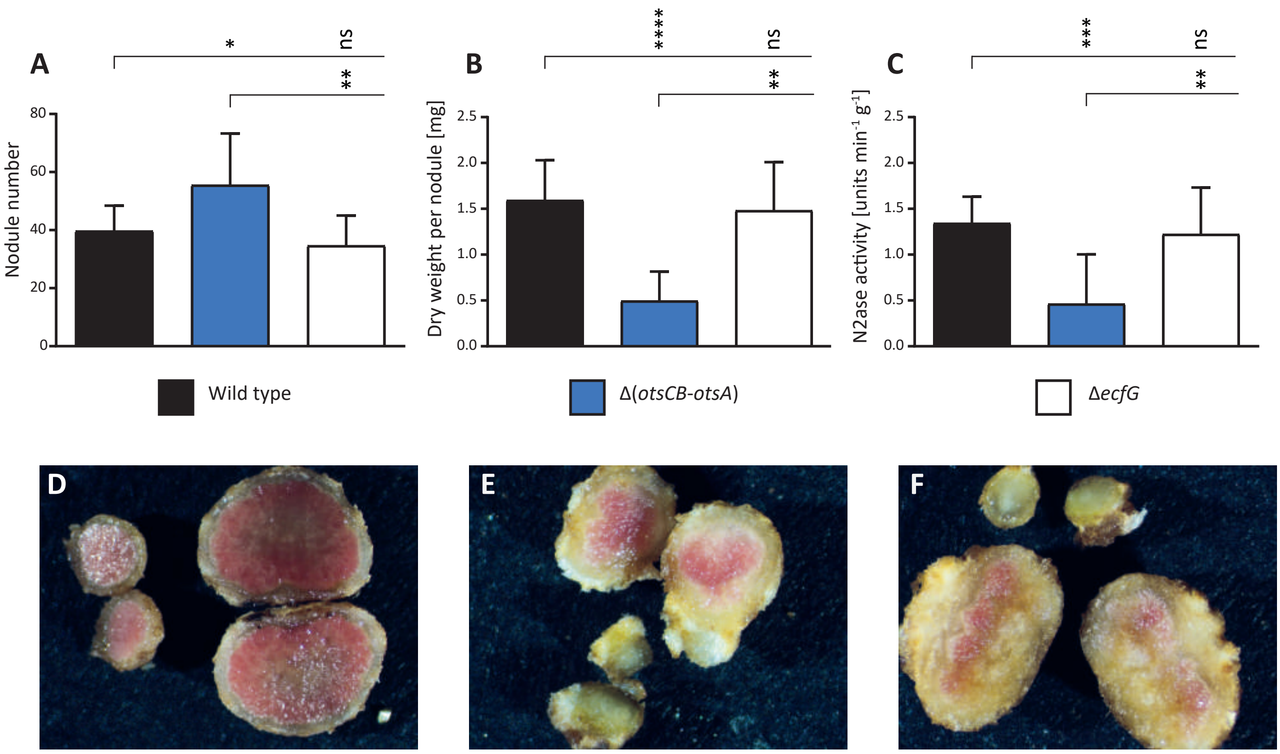

FIG. S8. Symbiotic phenotype of wild type, Δ(*otsCB-otsA*), and Δ*ecfG* mutants 28 dpi. Cells of B. *diazoefficiens* wild type (strain 110*spc*4), Δ(*otsCB-otsA*) (9871), and Δ*ecfG* (8404) were inoculated on soybean seedlings and harvested 28 dpi. Plants were evaluated for nodule number (A), dry weight per nodule (B), and nitrogenase activity measured by acetylene reduction (C). Cross sections of representative nodules showing overall nodule morphology and presence of reddish colour indicative for leghemoglobin (D, E, and F). n=10, displayed are means and error bars represent SD. Statistical significances of pairwise comparisons made between columns marked with a vertical tick and adjacent columns under horizontal lines were determined using one-way ANOVA with Šidák multiple comparison correction; ns P≥0.05, * P≤0.05, ** P≤0.01, *** P≤0.001, **** P≤0.0001.
