# Supplementary material for: Bradyrhizobium diazoefficiens Requires Chemical Chaperones To Cope with Osmotic Stress during Soybean Infection
Source: mBio. 2021 Mar 30;12(2):e00390-21. doi: 10.1128/mBio.00390-21 (PMC8092242; doi:10.1128/mBio.00390-21)
Supplement: TABLE S1 [file mBio.00390-21-st001.docx]

**Supplemental Tables S1A and S1B for**

***Bradyrhizobium diazoefficiens* requires chemical chaperones to cope with osmotic stress during soybean infection**

Raphael Ledermann, Barbara Emmenegger, Jean-Malo Couzigou, Nicola Zamboni, Patrick Kiefer, Julia A. Vorholt, and Hans-Martin Fischer

| **TABLE S1A.** Strains and plasmids used in this study | | |  |
| --- | --- | --- | --- |
| **Strain or plasmid** | **Relevant phenotype and genotype** | **Reference or source** | |
| Strains  *Escherichia coli* |  |  | |
| DH5α | *supE44* Δ*lacU169* (φ80 *lacZ*ΔM15) *hsdR17 recA1 gyrA96 thi-1 relA2* | BRL, Gaithers-burg, USA | |
| S17-1 λ*pir* | Sm^r^ Sp^r^ *hsdR* (RP4-2 *kan*::Tn*7 tet*::*Mu*; chromosomally integrated) | (1) | |
| *Bradyrhizobium diazoefficiens* | |  |  |
| 110*spc*4 | Sp^r^ wild type | (2) | |
| GusA-1 | Sp^r^ Tc^r^ (110*spc*4) P*_aphII_*-*gusA*, integrated ds *scoI* | (3) | |
| 8404 | Sp^r^ Km^r^ Δ*ecfG::aphII* | (4) | |
| OtsBA-1 | Sp^r^ Tc^r^ (110*spc*4) P*_aphII_*-*otsBA*, integrated ds *scoI* | This work | |
| 1687 | Sp^r^ Tc^r^(110*spc*4) P*_groESL2-mut1_*-*otsBA*, integrated ds *scoI* | This work | |
| 9987 | Sp^r^ Tc^r^ (110*spc*4) *gsmT-sdmT-metK2-ectABCD-ask*, integrated ds bll6649 | This work | |
| TreF-1 | Sp^r^ Tc^r^ (110*spc*4) P*_aphII_*-*treF*, integrated ds *scoI* | This work | |
| 8404-OtsBA-1 | Sp^r^ Km^r^ Tc^r^ (8404) Δ*ecfG::aphII* P*_aphII_*-*otsBA*, integrated ds *scoI* | This work | |
| 8404-1687 | Sp^r^ Km^r^ Tc^r^ (8404) Δ*ecfG::aphII* P*_groESL2-mut1_*-*otsBA*, integrated ds *scoI* | This work | |
| 9871 | Sp^r^ Sm^r^ (110*spc*4) Δ(*otsCB-otsA*)*::aadA* same orientation | This work | |
| 71-OtsBA-1 | Sp^r^ Sm^r^ Tc^r^ (9871) Δ(*otsCB-otsA*)*::aadA* P*_aphII_*-*otsBA*, integrated ds *scoI* | This work | |
| 71-1687 | Sp^r^ Sm^r^ Tc^r^ (9871) Δ(*otsCB-otsA*)*::aadA* P*_groESL2-mut1_*-*otsBA*, integrated ds *scoI* | This work | |
| 71-87 | Sp^r^ Sm^r^ Tc^r^ (9871) Δ(*otsCB-otsA*)*::aadA* *gsmT-sdmT-metK2-ectABCD-ask*, integrated ds bll6649 | This work | |
| 9885 | Sp^r^ Km^r^ (110*spc*4) Δ(*'glgX-treZ-treY*)*::aphII* same orientation | This work | |
| 9884 | Sp^r^ Tc^r^ (9885) Δ(*treS'-glgB-'glgX-*Δ[*glgX'-treZ-treY*]*::aphII*)*::tetA(C)* same orientation | This work | |
| 9864 | Sp^r^ Km^r^ (9884) Δ(bll6766-'*treS-*Δ[*treS'-glgB-glgX-treZ-treY*]*::tetA(C)*)*::aphII* same orientation | This work | |
| 9899 | Sp^r^ Tc^r^ (110*spc*4) Δbll0902*::tetA(C)* same orientation | This work | |
| 9904 | Sp^r^ Sm^r^ (110*spc*4) Δ*otsA::aadA* same orientation | This work | |
| 9904-GusA-1 | Sp^r^ Sm^r^ Tc^r^ (9904) Δ*otsA::aadA* P*_aphII_*-*gusA*, integrated ds *scoI* | This work | |
| 9905 | Sp^r^ (110*spc*4) Δ*otsC* | This work | |
| 9906_Sm | Sp^r^ Sm^r^ (110*spc*4) Δ*otsCB::aadA* same orientation | This work | |
| Plasmids |  |  | |
| pBluescript SK(+) | Ap^r^ cloning vector | Stratagene, La Jolla, CA, USA | |
| pGEM-T Easy | Ap^r^ PCR cloning vector | Promega, Ma-dison, WI, USA | |
| pBSL15Ω | Ap^r^ Sm^r^ Sp^r^ *aadA* donor plasmid | (5) | |
| pGEM-tetA(C) | Ap^r^ Tc^r^ *tetA(C)* donor plasmid | (6) | |
| pQH2 | Tc^r^ *tetAR cymR** vector | (7) | |
| pREDSIX | Ap^r^ mobilizable, high-copy-no. cloning and mutagenesis vector | (6) | |
| pRGD-KmR | Ap^r^ Km^r^ *aphII* donor plasmid | (6) | |
| pRGD-SmR | Ap^r^ Sp^r^ Sm^r^ *aadA* donor plasmid | (6) | |
| pRGD-TcR | Ap^r^ Tc^r^ *tetA(C)* donor plasmid | (6) | |
| pRJPaph-gfp | Tc^r^ P*_aphII_*-*gfp+* for genomic integration ds of *scoI* | (3) | |
| pRJPaph-gfp_a1 | Tc^r^ P*_aphII_*-*gfp+* for genomic integration ds of *scoI,* exchanged *Pst*I site ds of gfp+ with *Kpn*I and *Aat*II sites | (3) | |
| pRJPaph-gusA | Tc^r^ P*_aphII_*-*gusA* for genomic integration ds of *scoI* | (3) | |
| pRJPaph-lacZYA | Tc^r^ P*_aphII_*-*lacZYA* for genomic integration ds of *scoI* | (3) | |
| pRJ9937 | Tc^r^ *lacZYA* for integration as artificial operon ds of σ^EcfG^-controlled bll6649 | (8) | |
| pBSL15-tetA(C) | Ap^r^ Tc^r^ (pBSL15Ω) *tetA(C)* donor plasmid | This work | |
| pRJPaph-otsA | Tc^r^ (pRJPaph-gfp) P*_aphII_*-*otsA* for genomic integration ds of *scoI* | This work | |
| pRJPaph-otsB | Tc^r^ (pRJPaph-gfp) P*_aphII_*-*otsB* for genomic integration ds of *scoI* | This work | |
| pRJPaph-otsBA | Tc^r^ (pRJPaph-otsB) P*_aphII_*-*otsBA* for genomic integration ds of *scoI* | This work | |
| pRJPaph-treF | Tc^r^ (pRJPaph-gfp) P*_aphII_*-*treF* for genomic integration ds of *scoI* | This work | |
| pRJ1687 | Tc^r^ (pRJPaph-otsBA) P*_groESL2-mut1_*-*otsBA* for genomic integration ds of *scoI* | This work | |
| pRJ9871 | Ap^r^ Sm^r^ Sp^r^ (pREDSIX) *otsC* up- and *otsA* downstream regions separated by *aadA*, oriented from up- to downstream | This work | |
| pRJ9864 | Ap^r^ Km^r^ (pREDSIX) bll6766 up- and *treY* downstream regions separated by *aphII*, oriented from up- to downstream | This work | |
| pRJ9884 | Ap^r^ Tc^r^ (pRJ9864) *treS* internal fragment and *treY* downstream regions separated by *tetA(C)*, oriented from up- to downstream | This work | |
| pRJ9885 | Ap^r^ Km^r^ (pRJ9864) *glgX* internal fragment and *treY* downstream regions separated by *aphII*, oriented from up- to downstream | This work | |
| pRJ9899 | Ap^r^ Tc^r^ (pREDSIX) bll0902 up- and downstream regions separated by *tetA(C)*, oriented from up- to downstream | This work | |
| pRJ9903 | Ap^r^ (pGEM-T Easy) 'bll0324-*otsB*-*otsA* | This work | |
| pRJ9904 | Ap^r^ Sm^r^ Sp^r^ (pREDSIX) *otsA* up- and *otsA* downstream regions separated by *aadA*, oriented from up- to downstream | This work | |
| pRJ9905 | Ap^r^ Tc^r^ (pREDSIX) *otsC* up- and downstream regions flanked by *tetA(C)* | This work | |
| pRJ9906 | Ap^r^ Tc^r^ (pREDSIX) *otsC* up- and *otsB* downstream regions separated by *tetA(C)*, oriented from up- to downstream | This work | |
| pRJ9906_Sm | Ap^r^ Sm^r^ Sp^r^ (pRJ9906) *otsC* up- and *otsB* downstream regions separated by *aadA*, oriented from up- to downstream | This work | |
| pRJ9987 | Tc^r^ (pRJ9937) *gsmT-sdmT-metK2-ectABCD-ask* for integration ds of bll6649 | This work | |
| Abbreviations: Ap: ampicillin; Km: kanamycin; Sm: streptomycin; Sp: spectinomycin; Tc: tetracycline; ^r^: resistance; P: promoter with subscript indicating the associated gene; ds: downstream; strain or plasmid names indicated in brackets after antibiotic resistances refer to progenitor strains or plasmids. Same orientation refers to the orientation of the inserted antibiotic resistance gene relative to the deleted gene. | | |  |

**TABLE S2B.** Oligonucleotides used in this study

| Name | Sequence (5’-3’) |
| --- | --- |
| otsAB-1 | GCTTAATTAACGGCGCCTGAGAGG |
| otsAB-2 | AGGTACCAGCTCGCTCTGATCTTCC |
| otsAB-3 | AGGTACCGTCCGTTTCCGAAGG |
| otsAB-4 | GACTAGTCGGCTTCGGAAGAGG |
| otsAB-5 | TCGCAGATTGGCGACATAG |
| otsAB-6 | AAGGCCACCGACGAGATTTCC |
| otsAB-7 | AGCTAGCAGACGCTCGCCGTTGAGG |
| otsAB-8 | GACTAGTACGCGGTCGGCAC |
| otsAB-9 | GTTCCAATGCATAGCAGGAACGGCCTCCAAC |
| otsA-1 | CAGCGAGACGTTTCCTTTGG |
| otsA-2 | GGGCTAACTAGTAAACGGGGTGGAATGAACAG |
| otsA-3 | TGGAACCTGCAGTTGACGATCTCTTCCTTCCCC |
| otsA-4 | ACTAGTAAGAAGGAGATATAATTATGCTCAATCAGAAAATTCAAAACCC |
| otsB-1 | GCTGATGCGGTTGACGAG |
| 0324-1 | GGTTAACTAGTCAGGCATGATCGCGAACAC |
| 0324-2 | GAAAGCCAATTGTTTATGGAGTAGACTATGAAATCGG |
| 0324-3 | GGTTTGCAATTGACCGATCATCGGGGTGCGGCATTC |
| 0324-4 | ACAGAACTGCAGCCGAAAGCACCCGCCTCCG |
| 0324-5 | TCGGCGCATCGAAGTGACC |
| 0324-6 | TCGGCCAAGACCCTGTCATCC |
| 0324-7 | CACGCTTGTGCGTGATCC |
| 0324-8 | GCGCTGAAGCAAAGTCCC |
| 0902-1 | GATCGGCATGCATCAGCTC |
| 0902-2 | AGTGAACTAGTCCGTCGTCCTGGCGAAAG |
| 0902-3 | GGAATACTAGTGAGCCCGGGACCCATAGC |
| 0902-4 | GGTTCTCAATTGCGCGGTAACGGTTTCGTAGG |
| 0902-5 | AAGGCGCTGCCGAAAGGC |
| 0902-6 | AAGAAGCGCCCGGATGCAC |
| tre_-1 | GGTTAATTAAGGGATCGCCTCGAC |
| tre_-2 | CGGTACCTATAGAAAGCCGCTTGTG |
| tre_-3 | TGGTACCCACGACTTGCACCTTGAC |
| tre_-4 | GACTAGTTTCCAAGGCGTGGGAG |
| tre_-9 | ATTTCAGCGCCGCCATGTC |
| tre_-10 | ATCGGCGGCGAGACATTCTTC |
| tre_-12 | GCTCCGTTAATTAAGAAGCGCGACGACGTCCC |
| tre_-13 | GGTTCTCATATGTGCCTTCGCCGCTCCAGTAG |
| tre_-14 | GGTCCCTTAATTAACTGGTGGTGCCGGTCAAC |
| tre_-15 | GGTGAAACTAGTAGGTAGGGCGACTGCTCC |
| tre_-17 | GGCCAGCTCTATGGCTATCG |
| tre_-18 | CCTATGTCCGGCAGTACCAC |
| treF-1 | ACTAGTAAGAAGGAGATATAATTATGCTCAATCAGAAAATTCAAAACCC |
| treF-2 | CTGCAGCAGCAGGAAGTTAGCGGCTG |
| treF-3 | GGTGTGGTACGCCGTTTAATTG |
| PgroESL2-mut1_f | GTACCTTGTTTGCCGAGCCAAGCCGGCTTATTTTCGG |
| PgroESL2-mut1_r | CCGAAAATAAGCCGGCTTGGCTCGGCAAACAAG |
| Q1 | GGACTAGTAACAAACAGACAATCTGGTCTGTTTGTACC |
| Q2 | GGGAAGATCTTACAAACAGACCAGATTGTCTGTTTGTTCCGAAAATAAG |
| GSDMT-3 | AAGATCTAACCATTCGCCAAGCTGAG |
| GSDMT-4 | GACTAGTGGCCAATTGGATCACCCTTTGCGGAAG |
| SAM-3 | CAGATCTCCGGCAATTGGCTCGAATAAACTATAGCAAAGG |
| SAM-4 | CACTAGTTTGGAATTCTGTTACTTAAGGCCAGCTGC |
| ect3-1 | CCTTAAGTTTGAGTTTCCCCACCGTAATG |
| ect3-2 | CCAATTGCGTTGACTAGTTCAGACGGTTTCGGCCTCCAG |
| ectD-1 | GGCTAGCAAGGAGGTAAGCGTGAACCC |
| ectD-2 | GGGTACCGCCCAATTGCCTCAGAGATACTGTTGC |
| ask-1 | AAGATCTTCGCCTTAAGGGCCCAATTGGATTCGGACGCTATAAAAAG |
| ask-2 | TGGTACCCGAACTAGTTCAGGCCGCGGCAATCAC |
| GSMT-1 | TGGAAGCCGGTACCTGTTG |
| 6649-3 | CGCACTCCTGATCGACTACAAG |
| RT-1 | CAGATCCAGCGTCGCAATCG |
| RT-3 | GATGAACGCCAGTTAAACGG |
| RT-4 | TTCGATGCGCCGAGCGATGT |
| RT-5 | GCGAAGATCAAGTCGATGTC |
| RT-7 | GAACGCAAATCGCTCGAAC |
| gfp+-4 | AGATATTCCGGGCGGATTTC |
| gusA-3 | AGCAGGGAGGCAAACAATG |
| Kan-2 | CATCGCCTTCTATCGCCTTC |
| Kan-4 | CAATAGCAGCCAGTCCCTTC |
| Strp-3 | GCCTTGATGTTACCCGAGAG |
| Strp-5 | GCGAAGTAATCGCAACATCC |
| tetA(C)-1 | GTGCCGAGGATGACGATGAG |
| tetA(C)-2 | CGCCCTATACCTTGTCTGCC |

**References**

1. de Lorenzo V, Cases I, Herrero M, Timmis KN. 1993. Early and late responses of TOL promoters to pathway inducers: Identification of postexponential promoters in *Pseudomonas putida* with *lacZ-tet* bicistronic reporters. J Bacteriol 175:6902-7.

2. Regensburger B, Hennecke H. 1983. RNA polymerase from *Rhizobium japonicum*. Arch Microbiol 135:103-109.

3. Ledermann R, Bartsch I, Remus-Emsermann MN, Vorholt JA, Fischer HM. 2015. Stable fluorescent and enzymatic tagging of *Bradyrhizobium diazoefficiens* to analyze host-plant infection and colonization. Mol Plant Microbe Interact 28:959-67.

4. Gourion B, Sulser S, Frunzke J, Francez-Charlot A, Stiefel P, Pessi G, Vorholt JA, Fischer HM. 2009. The PhyR-σ^EcfG^ signalling cascade is involved in stress response and symbiotic efficiency in *Bradyrhizobium japonicum*. Mol Microbiol 73:291-305.

5. Lindemann A, Koch M, Pessi G, Muller AJ, Balsiger S, Hennecke H, Fischer HM. 2010. Host-specific symbiotic requirement of BdeAB, a RegR-controlled RND-type efflux system in *Bradyrhizobium japonicum*. FEMS Microbiol Lett 312:184-91.

6. Ledermann R, Strebel S, Kampik C, Fischer HM. 2016. Versatile vectors for efficient mutagenesis of *Bradyrhizobium diazoefficiens* and other Alphaproteobacteria. Appl Environ Microbiol 82:2791-9.

7. Kaczmarczyk A, Vorholt JA, Francez-Charlot A. 2013. Cumate-inducible gene expression system for sphingomonads and other Alphaproteobacteria. Appl Environ Microbiol 79:6795-802.

8. Ledermann R, Bartsch I, Müller B, Wülser J, Fischer HM. 2018. A functional general stress response of *Bradyrhizobium diazoefficiens* is required for early stages of host plant infection. Mol Plant Microbe Interact 31:537-547.
