# Supplementary material for: Bradyrhizobium diazoefficiens Requires Chemical Chaperones To Cope with Osmotic Stress during Soybean Infection
Source: mBio. 2021 Mar 30;12(2):e00390-21. doi: 10.1128/mBio.00390-21 (PMC8092242; doi:10.1128/mBio.00390-21)
Supplement: TEXT S1 [file mBio.00390-21-s0001.docx]

**Supplemental material for**

***Bradyrhizobium diazoefficiens* requires chemical chaperones to cope with osmotic stress during soybean infection**

Raphael Ledermann, Barbara Emmenegger, Jean-Malo Couzigou, Nicola Zamboni, Patrick Kiefer, Julia A. Vorholt, and Hans-Martin Fischer

**Plasmid and strain constructions**

For deletion of the *otsCB-otsA* region (Fig. S1A), plasmid pRJ9871 was constructed by PCR amplification of the *otsA* downstream (otsAB-1/-2) and *otsC* upstream regions (otsAB-3/-4) and cloning them into pREDSIX using *Pac*I and *Spe*I (on the otsAB-3/-4 amplified fragment) and inserting an *aadA* streptomycin resistance released with *Kpn*I from pRGD-SmR into the unique *Kpn*I site. Correct deletion was verified by PCR using primer pairs otsAB-5/Strp-5 and Strp-3/otsAB-6. For detailed functional analysis of the *ots* region, individual genes were deleted. For deletion of *otsA*, a 2 kb fragment containing the 5' end of *otsA*, *otsB,* and the 3' end of *otsC* was PCR amplified using primers otsAB-7 and otsAB-8 and cloned into pGEM-T easy, generating plasmid pRJ9903. From this plasmid, the *otsA* upstream region was released using *Spe*I and the endogenous *Nsi*I site located in the *otsB-otsA* intergenic region. The *Nsi*I site was blunted using the Klenow fragment of *E. coli* DNA polymerase I. From pRJ9871, the *otsC* upstream region was released using *Xba*I and *Eco*RI (blunted using the Klenow fragment enzyme) and replaced by the *otsA* upstream fragment, generating pRJ9904. Correct deletion of *otsA* was verified by PCR using primer pairs otsAB-5/Strp-5 and Strp-3/otsA-1.

For deletion of the *otsCB* operon, we amplified the *otsC* upstream region with primers otsAB-9 and otsAB-4 and cut the PCR amplified fragment *Nsi*I and *Eco*RI. The *otsB* downstream fragment was excised from pRJ9903 using *Nhe*I and *Nsi*I and the two flanking regions were cloned into *Nhe*I- and *Mun*I-digested pREDSIX. First, a tetracycline resistance cassette (released with *Pst*I from pBSL15-tetA(C) was inserted into the unique *Nsi*I site, yielding plasmid pRJ9906. The tetracycline resistance was then replaced with a streptomycin resistance (by *Kpn*I digestion), generating plasmid pRJ9906_Sm. Correct mutation was verified by PCR using primer pairs otsB-1/Strp-5 and Strp-3/otsAB-6. For markerless deletion of *otsC*, the *otsC* downstream region was amplified using primers 0324-1/-2 and the upstream region was amplified with primers 0324-3/-4. Notably, the first and last three coding triplets and the endogenous *otsC* stop codon were retained in the amplicons to end up with a markerless in-frame deletion. The two fragments were fused via a *Mun*I site (generating two artificial in-frame codons for Gln and Leu between the first and last three codons of *otsC*) and ends for subsequent cloning were prepared by *Pst*I and *Spe*I digestion followed by ligation into *Xba*I- and *Nsi*I-linearized pREDSIX backbone. A pRGD-TcR-derived (*Spe*I) tetracycline resistance cassette was cloned into the *Nhe*I site aside the two flanking regions to generate pRJ9905. Correct mutant genotype was verified by PCR using primer pairs 0324-5/-6 (amplifying flanking regions and the desired deletion or *otsC* in case of the wild type) and 0324-7/-8 (amplifying an *otsC*-internal fragment expected to be deleted in the mutant).

For deletion of bll0902 (Fig. S1B), flanking regions were amplified using primer pairs 0902-1/-2 and 0902-3/-4. Flanking regions were cloned in pREDSIX using *Nsi*I and *Mun*I sites and generating a unique *Spe*I site. Tetracycline resistance cassette from pRGD-TcR was released by *Spe*I digestion and cloned between the two flanking regions, yielding plasmid pRJ9899. Correct mutants were verified by PCR using primer pairs tetA(C)-1/0902-6 and tetA(C)-2/0902-5.

For mutagenesis of the *treZY* region (bll6766-*treS-glgB-glgX-treZ-treY*) (Fig. S1C), plasmid pRJ9864 was constructed. The bll6766 upstream region (primer pair tre_-1/-2) and the *treY* downstream region (primer pair tre_-3/-4) were amplified and cloned in pREDSIX using *Pac*I in combination with either *Spe*I (for the tre_-3/-4 amplified region) or *Nhe*I (for pREDSIX). The unique site *Kpn*I in the resulting plasmid was used to insert a kanamycin resistance cassette released from pRGD-KmR. Because direct deletion of the entire bll6766-*treS-glgB-glgX-treZ-treY* region (14'174 bp) was not successful in one step, it was deleted sequentially. First, we constructed plasmid pRJ9885 by excising the bll6766 upstream region in pRJ9864 (*Nde*I and *Pac*I) and replacing it with a *glgX*-internal fragment amplified with the primer pair tre_-12/-13. The resulting plasmid was used to delete the 3' end of *glgX* and *treZY* generating strain 9885. Correct deletion was verified by PCR using primer pairs tre_-17/Kan-4 and Kan-2/tre_-10. Next, plasmid pRJ9884 was constructed by releasing the bll6766 upstream region and *aphII* from pRJ9864 (*Pac*I and *Spe*I) and inserting a PCR-amplified, *treS*-internal fragment (primer pair tre_-14/-15). The unique *Spe*I site was used for insertion of a *tetA(C)* cassette released from pRGD-TcR resulting in plasmid pRJ9884. Using this plasmid, strain 9885 was further mutated by deletion of the 3' end of *treS*, *glgB*, the remaining 5' end of *glgX* and the *aphII* kanamycin resistance gene, generating strain 9884. Correct deletion was verified by PCR using primer pairs tre_-18/tetA(C)-1 and tetA(C)-2/tre_-10. Finally, strain 9884 was used to delete the remaining part of the *treZY* region (bll6766, the 5' end of *treS* and *tetA(C)*) using plasmid pRJ9864. Correct deletion was PCR verified using primer pairs Kan-4/tre_-9 and Kan-2/tre_-10*.*

For construction of the *otsBA* complementation constructs, *otsA* was amplified using primers otsA-2/-3, cut with *Pst*I and *Spe*I and cloned into identically digested pRJPaph-gfp, generating pRJPaph-otsA. For plasmid pRJPaph-otsB, *otsB* was excised from pRJ9903 (*Pst*I and *Not*I, blunted with Klenow enzyme) and ligated with pRJPaph-gfp (*Pst*I and *Spe*I, blunted with Klenow enzyme). Since a hairpin structure is present downstream of *otsA*, we suspected that it could act as transcriptional terminator and decided to generate an artificial P*_aphII_*-*otsB*-*otsA* operon for simultaneous constitutive expression of both genes. To do so, we released *otsA* and the homologous region for integration downstream of *scoI* (1) from pRJPaph-otsA (*Xba*I and *Bgl*II, blunted with Klenow enzyme) and inserted the fragment downstream of *otsB* in pRJPaph-otsB (*Xba*I and *Pst*I, blunted with Klenow enzyme), resulting in pRJPaph-otsBA. The *groESL*_2_-derived promoter was assembled using two oligos (PgroESL2-mut1_f and PgroESL2-mut1_r) and cloned into *Hpa*I- and *Bsr*GI-digested pQH2 (2), from where it was amplified using primers Q1 and Q2. The product was digested with *Spe*I (blunted with Klenow enzyme) and *Bgl*II. To exchange the *aphII* promoter with a weaker *groESL*_2_-derived promoter, P*_aphII_* was removed using *Bgl*II and *Sca*I from a pRJPaph-otsBA derivative and exchanged with the PCR product prepared as described above, resulting in pRJ1687.

Similarly, for expression of the *E. coli treF* gene, encoding a cytoplasmic trehalase, we amplified *treF* from genomic *E. coli* DNA using primers treF-1/-2. Notably, treF-1 included a 5' extension representing a ribosomal binding site optimized for *B. diazoefficiens*. The fragment was cut with *Spe*I and *Pst*I and ligated into identically pretreated pRJPaph-gfp, generating pRJPaph-treF.

Plasmids were mobilized into *B. diazoeffciens* by biparental mating and exconjugants were tested by PCR using primer gfp+-4 in combination with otsA-4 (for pRJPaph-otsBA integration) or treF-3 (for pRJPaph-treF integration).

Constructs for integration of glycine betaine and (hydroxy)ectoine biosynthesis genes downstream of the σ^EcfG^-controlled gene bll6649 (3, 4) were sequentially assembled into a pRJ9937 derivative containing bll6649 for homologous recombination, resulting in a bll6649-*gsmT*-*sdmT*-*metK2*-*ectABCD*-*ask* operon upon chromosomal integration. Primer pairs GSDMT-3/-4 and SAM-3/-4 were used to amplify the *gsmT*-*sdmT* operon and *metK2*, respectively, from genomic DNA of *Halorhodospira halochloris* DSM 1059 (DSMZ). Primer pairs ect3-1/-2, ectD‑1/-2, and ask-1/-2 were used to amplify the *ectABC* operon, *ectD*, and *ask*, respectively, from genomic DNA of *Pseudomonas stutzeri* ATCC 17588 (DSMZ). Correct integration of the final plasmid pRJ9987 into *B. diazoefficiens* strains 110*spc*4 (wild type) and 9871 (Δ(*otsCB-otsA*)) was verified with primer pair GSMT-1 and 6649-3. Strains were GusA-tagged as described before (1).

**Legends of supplemental figures**

**FIG. S1.** Genetic map of three *B. diazoefficiens* loci encoding trehalose biosynthesis genes and genotype of respective deletion mutants. Mutant strain numbers are indicated below the deleted regions or the inserted antibiotic resistance gene cassettes. Locus encoding genes for trehalose biosynthesis via the T6P pathway (A): *otsA* (bll0322) encoding trehalose-6-phosphate synthase (dark blue), *otsB* (bll0323) encoding trehalose-6-phosphate phosphatase (light blue), *otsC* (bll0324) encoding an MFS-like sugar transport protein which putatively mediates trehalose uptake (purple). Constructed mutants lack either the entire locus (Δ(*otsCB-otsA*)::*aadA*; strain 9871), *otsA* (Δ*otsA*::*aadA*; 9904), *otsC* and *otsB* (Δ*otsCB*::*aadA*; 9906_Sm), or *otsC* (Δ*otsC* markerless in-frame deletion; 9905). The bll0902 (light green) locus encoding a putative trehalose synthase which converts maltose into trehalose (B). In mutant strain 9899 (Δbll0902::*tetA*(*C*)), bll0902 is deleted. Locus encoding the putative operon for trehalose biosynthesis genes via the TreS and the TreZY pathways (C): *treS* (blr6767; dark green), *treZ* (blr6771; yellow), *treY* (blr6770; orange) coding for trehalose synthase, malto-oligosyl trehalose trehalohydrolase and malto‑oligosyl trehalose synthase, respectively, and three genes related to glycogen metabolism: blr6766 (putative glucanohydrolase) *glgB* (blr6768; putative 1,4‑α-glucan branching enzyme) and *glgX* (blr6769; putative glycogen debranching enzyme). Generated mutants carry deletions of the entire gene cluster (Δ(blr6766-*treS-glgB-glgX-treZ-treY*)::*aphII*; strain 9864) or *treZY* and the 3’ end of *glgX* (Δ(*glgX*’-*treZY*)::*aphII*; 9885). Maps are drawn to scale with the upper scale bar applying to panels A and B and the lower to panel C.

**FIG. S2.** Symbiotic phenotype of additional mutants in trehalose biosynthesis and related genes. Cells of *B. diazoefficiens* wild type (strain 110*spc*4), Δ*otsCB* (9906_Sm), Δ*otsC* (9905), Δbll0902 (9899), Δ(*glgX'-treZY*) (9985), and Δ(blr6766-*treS-glgBX-treZY*) (9964) were inoculated on soybean seedlings and harvested 21 dpi (n≥9). Plants were evaluated for nodule number (A, F, and L), dry weight per nodule (B, G, and M), and nitrogenase activity measured by acetylene reduction (C, H, and N). Cross sections of representative nodules showing overall nodule morphology and presence of reddish colour indicative for leghemoglobin (D, E, I, J, O, P, Q, and R). Number of infection threads formed by a GusA-tagged Δ*otsA* mutant compared to a GusA-tagged wild type (GusA-1) at 5, 8, and 11 dpi (K). Displayed are means and error bars represent SD (n=5). Statistical significances of pairwise comparisons made between columns marked with a vertical tick and adjacent columns under horizontal lines were determined using a two-tailed Student’s *t* test (A, B, C, F, G, and H) or one-way ANOVA with Šidák multiple comparison correction (K, L, M, and N); ns *P*≥0.05, * *P*≤0.05, ** *P*≤0.01, **** *P*≤0.0001.

**FIG. S3.** Trehalose synthesis via the T6P pathway cannot fully restore symbiotic competitiveness and stress tolerance of the Δ*ecfG* mutant. (A) Mixtures of the *B. diazoefficiens* Δ(*otsCB*-*otsA*) and Δ*ecfG* mutant strains, both complemented with constitutively expressed *otsBA* genes (strain 71-1687 and 8404-1687, respectively), containing a total of approx. 15'000 cells at three different ratios (71-1687 : 8404-1687 = 43% : 57% (left stacked bar), 70% : 30% (middle), 16% : 84% (right); dashed horizontal lines) were inoculated on soybean seedlings. The nodules of six individual plants per inoculum were harvested 19 dpi and crushed with a plastic pistil in a 2-ml Eppendorf tube. Aliquots of serial dilutions of the resulting 18 extracts were plated in parallel on PSY agar plates selective for one of the two strains. Colony-forming units (CFUs) were counted and the relative abundance of both strains in the nodules of the six individual plants was calculated. Significance of differences detected by one-sample Student’s *t* tests between ratios in the inocula and in reisolated bacteroids is indicated above the stacked bars. *** *P* ≤0.001, **** *P* ≤0.0001.

Cells of *B. diazoefficiens* wild type (strain 110*spc*4), Δ*ecfG*; (8404), and strains harboring constitutively expressed *otsBA* genes in the wild-type (1687), Δ*ecfG* (8404-1687), and Δ(*otsCB*-*otsA*) (71-1687) background were grown to mid-exponential phase in V3C medium, adjusted to an OD_600_=0.1, and 4-µl aliquots of serial dilutions were spotted on V3C agar plates imposing either osmostress (B), salt stress (C), or alkaline pH stress (D).

**FIG. S4.** Trehalose content of *B. diazoefficiens* wild-type and mutant cells grown under stressed and unstressed conditions. Cells of the wild type (strain 110*spc*4), or Δ*ecfG* mutant (8404), Δ(*otsCB-otsA*) (9871), Δ*otsA* (9904), Δ*otsCB* (9906_Sm), Δ(*glgX'-treZY*) (9985), Δ(blr6766-*treS-glgBX-treZY*) (9964), Δbll0902 (9899), and of a wild type-derived strain expressing *E. coli treF* from a constitutive promoter (TreF-1) were grown in PSY medium and transferred either to fresh PSY (unstressed) or V3 minimal medium lacking a carbon source (stressed) and incubated for a further 12 h. Cells were harvested, washed, extracted, and trehalose content was measured as described in Materials and Methods. Relative trehalose content is indicated as ion count (IC). n=6, displayed are means and error bars represent SD. Statistical significances of pairwise comparisons listed in the table below the graph were determined using one-way ANOVA with Šidák multiple comparison correction; ns *P*≥0.05, * *P*≤0.05, **** *P*≤0.0001.

**FIG. S5.** Exogenous trehalose can rescue a Δ*otsA* mutant under hyperosmotic conditions and in symbiosis. *B. diazoefficiens* wild type (strain 110*spc*4), Δ*ecfG* (8404), Δ(*otsCB-otsA*) (9871), Δ*otsA* (9904), Δ*otsCB* (9906_Sm), and a strain expressing *E. coli treF* from a constitutive promoter in a wild-type background (treF-1) were adjusted to OD_600_=0.01. Strains then were streaked on PSY agar plates containing a sorbitol gradient ranging from 0 mM to 700 mM and supplemented without (center) or with 5 mM trehalose (right). No sorbitol was added to the control plate (left) (A). Reduced growth of the Δ*otsCB* mutant likely is due to increased trehalose-6-phosphate levels resulting in sugar phosphate toxicity (see (5) and references therein). For evaluating symbiotic phenotypes, wild-type (strain 110*spc*4) or Δ*otsA* mutant (9904) cells were inoculated on soybean seedlings. These were grown in 180-ml jars filled with vermiculite which was soaked with mineral salts solution and supplemented with 10 mmoles of the indicated sugar or no additional sugar (for details, see Materials and Methods). Plants were harvested 21 dpi and evaluated for nodule number (B), dry weight per nodule (C), and nitrogenase activity measured by acetylene reduction (D). Cross sections of representative nodules showing overall nodule morphology and presence of reddish color indicative for leghemoglobin (E). Displayed are means and error bars represent SD (n≥8). Statistical significances of pairwise comparisons made between columns marked with a vertical tick and adjacent columns under horizontal lines were determined using one-way ANOVA with Šidák multiple comparison correction; ns *P*≥0.05, * *P*≤0.05, ** *P*≤0.01, *** *P*≤0.001, **** *P*≤0.0001. Note that the data shown in panels B, C, and D, and E of this figure were generated in the same experiment underlying the data set shown in Fig. 3. Hence, the same data are shown for the reference conditions (wild type and Δ*otsA* mutant, with and without trehalose) in both figures. Statistical analysis was performed on the entire data set.

**FIG. S6.** Transcriptional organisation and regulation of the *B. diazoefficiens otsCB-otsA* gene region. Cells of the wild type (strain 110*spc*4) and Δ*ecfG* mutant (8404) were grown in PSY medium (unstressed, U) or PSY medium containing 30 mM NaCl (stressed, S). Total RNA was extracted and cDNA synthesized using random hexameric primers. Genetic organization and primer binding sites (A) used for endpoint RT-PCR resulting in the amplification products shown in (B). *otsC* and *otsB* form an operon while *otsA* is transcribed separately. Unlike *otsCB*, transcription of *otsA* is only partially dependent on σ^EcfG^.

**FIG. S7.** Complementation of trehalose biosynthesis mutants by expression of recombinant genes encoding biosynthesis for different chemical chaperones. The chaperone biosynthesis genes were integrated as an artificial operon downstream of the σ^EcfG^-controlled gene bll6649. Genes for glycine betaine biosynthesis (*gsmT* and *sdmT*; dark blue) and the methyl donor (SAM)-regenerating enzyme MetK2 (light blue) were derived from *Halorhodospira halochloris*. Genes for ectoine (*ectABC*; dark green), hydroxyectoine (*ectD*; bright green) and a feedback-insensitive aspartate kinase (*ask*; light green) were derived from *Pseudomonas stutzeri*. Both gene clusters including the accessory function genes *metK2* and *ask* are present in the same order in the respective host organism (A). Biosynthesis of glycine betaine. GsmT (glycine/sarcosine methyltransferase) and SdmT (sarcosine/dimethylglycine methyltransferase) successively N‑methylate glycine using SAM as a methyl donor. Notably, this pathway differs from many other glycine betaine-producing organisms, which rather employ oxidation of choline (B). Biosynthesis of ectoine and hydroxyectoine. Biosynthesis starts from aspartate-4-semialdehyde which is aminated to 2,4-diaminobutanoate by EctB using glutamate as an aminodonor. EctA transfers the acetyl group from acetyl-CoA to the γ-amino group forming N4-acetyl-2,4-diaminobutanoate which in turn is cyclized to ectoine by EctC. Hydroxylation of ectoine to hydroxyectoine by EctD employs molecular oxygen, and α-ketoglutarate as an additional electron donor. Biosynthesis of aspartate‑4‑semialdehyde, which is also an intermediate in homoserine and lysine biosynthesis, involves the endogenous enzymes LysC and Asd (black) (C). Cells of wild type (strain 110*spc*4), Δ(*otsCB*-*otsA*) (9871), and the respective backgrounds expressing the heterologous biosynthesis operon *gsmT*-*sdmT*-*metK2*-*ectABCD*-*ask* (GSMABCDA; strains 9987 and 71-87) were grown to mid-exponential phase in PSY before the σ^EcfG^-dependent bll6649 promoter located upstream of the operon was induced by addition of 40 mM NaCl. After further incubation for 5 h, cell extracts were prepared and chemical chaperones were determined by LC-MS measurements as described in Materials and Methods (D). Displayed are means and error bars represent SD (n=5). Statistical significances of pairwise comparisons made between columns marked with a vertical tick and adjacent columns under horizontal lines were determined for each individual compound using one-way ANOVA with Šidák multiple comparison correction; ns *P*≥0.05, * *P*≤0.05, *** *P*≤0.001, **** *P*≤0.0001. Suspensions of the indicated strains grown identically as described for (D) were adjusted to an OD_600_=0.1, serially diluted and then 4 µl aliquots were spotted on PSY agar plates with the indicated ionic (NaCl) and non-ionic (sorbitol) osmostress (E).

**FIG. S8.** Symbiotic phenotype of wild type, Δ(*otsCB*-*otsA*), and Δ*ecfG* mutants 28 dpi. Cells of *B. diazoefficiens* wild type (strain 110*spc*4), Δ(*otsCB*-*otsA*) (9871), and Δ*ecfG* (8404) were inoculated on soybean seedlings and harvested 28 dpi. Plants were evaluated for nodule number (A), dry weight per nodule (B), and nitrogenase activity measured by acetylene reduction (C). Cross sections of representative nodules showing overall nodule morphology and presence of reddish colour indicative for leghemoglobin (*D*, *E*, and *F*). n=10, displayed are means and error bars represent SD. Statistical significances of pairwise comparisons made between columns marked with a vertical tick and adjacent columns under horizontal lines were determined using one-way ANOVA with Šidák multiple comparison correction; ns *P*≥0.05, * *P*≤0.05, ** *P*≤0.01, *** *P*≤0.001, **** *P*≤0.0001.

**SUPPLEMENTAL REFERENCES**

1. Ledermann R, Bartsch I, Remus-Emsermann MN, Vorholt JA, Fischer HM. 2015. Stable fluorescent and enzymatic tagging of *Bradyrhizobium diazoefficiens* to analyze host-plant infection and colonization. Mol Plant Microbe Interact 28:959-67.

2. Kaczmarczyk A, Vorholt JA, Francez-Charlot A. 2013. Cumate-inducible gene expression system for sphingomonads and other Alphaproteobacteria. Appl Environ Microbiol 79:6795-802.

3. Gourion B, Sulser S, Frunzke J, Francez-Charlot A, Stiefel P, Pessi G, Vorholt JA, Fischer HM. 2009. The PhyR-σ^EcfG^ signalling cascade is involved in stress response and symbiotic efficiency in *Bradyrhizobium japonicum*. Mol Microbiol 73:291-305.

4. Ledermann R, Bartsch I, Müller B, Wülser J, Fischer HM. 2018. A functional general stress response of *Bradyrhizobium diazoefficiens* is required for early stages of host plant infection. Mol Plant Microbe Interact 31:537-547.

5. Hubloher JJ, Zeidler S, Lamosa P, Santos H, Averhoff B, Muller V. 2020. Trehalose-6-phosphate mediated phenotypic change in *Acinetobacter baumannii*. Environ Microbiol doi:doi: 10.1111/1462-2920.15148.
